# Lifestyle changes in patients with non-alcoholic fatty liver disease: A systematic review and meta-analysis

**DOI:** 10.1371/journal.pone.0263931

**Published:** 2022-02-17

**Authors:** Tiziana Fernández, Macarena Viñuela, Catalina Vidal, Francisco Barrera

**Affiliations:** 1 Departamento Ciencias de la Salud, Facultad de Medicina, Pontificia Universidad Católica de Chile, Santiago, Chile; 2 Departamento de Gastroenterología, Facultad de Medicina, Pontificia Universidad Católica de Chile, Santiago, Chile; 3 Departamento de Ortopedia y Traumatología, Facultad de Medicina, Pontificia Universidad Católica de Chile, Santiago, Chile; Medizinische Fakultat der RWTH Aachen, GERMANY

## Abstract

**Background:**

Non-alcoholic fatty liver disease is a liver condition that is increasing worldwide and expected to become the number one cause of cirrhosis and hepatocellular carcinoma in the next 5 years. Currently there are no successful or approved pharmacological treatments. Weight loss is the first-line therapy as a 7 to 10% reduction improves steatosis, inflammation, hepatocyte ballooning, and fibrosis. To achieve this, lifestyle interventions including daily exercise and diet must be encouraged. We aimed to assess the effects of diet, exercise, or a combination of both compared to conventional treatment in patients with non-alcoholic fatty liver disease.

**Methods and finding:**

A literature search was performed in CENTRAL, EMBASE, and PubMed. Randomized controlled trials comparing lifestyle changes with conventional treatment were included, without date restriction. Two authors searched studies according to eligibility criteria, extracted data, and assessed study quality. Subgroup analysis was made by type of intervention, duration of intervention and supervision. We calculated mean differences between the intervention and the control group with their corresponding 95% confidence intervals. Quality of the evidence was assessed using the Cochrane Risk of bias tool. This study is registered in PROSPERO, number CRD42020184241, and checked with the PRISMA checklist. 30 RCTs met the inclusion criteria. Compared to conventional treatment, combined exercise with diet seems to elicit greater reductions in ALT (MD: -13.27 CI 95% -21.39, -5.16), AST (MD: -7.02 CI 95% -11.26, -2.78) and HOMA-IR (MD: -2.07 CI 95% -2.61, -1.46) than diet (ALT MD: -4.48 CI 95% -1.01, -0.21; HOMA-IR MD: -0.61 CI 95% -1.01, -0.21) and exercise (ALT and AST non-significant; HOMA-IR MD = -0.46 CI 95% -0.8, -0.12) alone. Additionally, exercise improved quality of life, cardiorespiratory fitness, and weight (MD: -2.64 CI 95% -5.18, -0.09).

**Conclusion:**

Lifestyle changes are effective in the treatment of NAFLD. Diet and exercise combined are superior to these interventions alone in improving liver enzymes and HOMA-IR.

## Introduction

NAFLD is a condition that includes a broad spectrum of liver diseases ranging from simple steatosis in which lipid droplets accumulate within the cytoplasm in over 5% of hepatocytes, to non-alcoholic steatohepatitis (NASH), which is characterized by steatosis, inflammation and hepatocyte cell ballooning frequently associated with fibrosis not explained by other factors [1] NAFLD is strongly associated with obesity, insulin resistance (IR), type 2 diabetes mellitus (T2DM) and dyslipidemia [2].

Its prevalence is approximately 25% worldwide and its incidence is increasing rapidly. It is expected to become the first cause of progression to cirrhosis and hepatocellular carcinoma in the next 5 years [3].

Lifestyle interventions are the first line therapy for NAFLD [4]. In most interventions, the main goal is between 7 to 10% weight loss as it improves steatosis, inflammation, hepatocyte ballooning and fibrosis [5].

Lifestyle changes include exercise and diet therapy [6]. Exercise has a large effect in intrahepatic triglycerides (IHL), enzyme levels, insulin sensitivity, glucose homeostasis [7, 8], free fat acid transportation and oxidation, radical oxygen production and inflammation [9]. Current guidelines for management of NAFLD recommend 150 minutes per week of moderate exercise [2] similar to the American College of Sports Medicine recommendations [10]. In terms of diet, the AASLD guideline recommends that decreasing caloric intake between 750–1000 kcal/day or 30% of the regular intake improves IR and hepatic steatosis [11], improving fasting glucose, IR, and alanine-transferase [12] and reducing free fatty acids. The combination of both interventions has demonstrated improvements in liver histology and liver enzymes [7] and is associated with weight reductions of 4.2% to 10.6% [13].

There are several publications regarding lifestyle changes and NAFLD reduction. However, only a few are systematic reviews with meta-analysis that study the impact of diet, exercise or both interventions together in NAFLD parameters. We aimed to assess the effect of diet, exercise, or the combination of both compared to conventional treatment in patients with NAFLD.

## Methods

### Protocol and registration

Our protocol was registered on PROSPERO (CRD42020184241). This protocol was designed and reported in line with Preferred Reporting Items for Systematic Reviews and Meta-Analyses Protocols (PRISMA-P) (S1 File) [14].

### Criteria for considering studies for this review

#### Studies included

We selected randomized clinical trials. Quasi-randomized, observational studies, pilot studies and studies evaluating the effects on animal models or in vitro conditions were excluded. We included studies in English and Spanish, and there were no restrictions based on publication status or date.

#### Population

Participants older than 18 years with different degrees of NAFLD including NASH, diagnosed based on liver biopsy, ultrasound (US), magnetic resonance imaging (MRI) or proton magnetic resonance spectroscopy (H-MRS) and with increased aspartate transferase (AST), alanine transferase (ALT) or gamma-glutamyl transpeptidase (GGT) [4, 11]. Steatohepatitis was defined as NAFLD activity score ≥ 5 [15] whereas significant fibrosis was considered as the F1-F2 stage or NAFLD fibrosis score < -1.455. Alcohol consumption thresholds were considered less than 20 g/day in women and >30 g/day in men. Studies include cirrhosis, defined as F4 stage or a NAFLD fibrosis score > 0.675, hepatocellular carcinoma, other liver conditions such as hepatitis, vascular disorders, drugs or toxin induced liver injuries or alcoholic liver disease [3]. Studies based on subjects with metabolic syndrome (defined as waist circumference > 102 cm in men and > 88 cm in women, triglycerides ≥ 150 mg/dl, HDL cholesterol < 40 mg/dl in men and < 50 mg/dl in woman, blood pressure ≥130/≥85 and fasting glucose ≥ 110 mg/dl) [16], obesity or T2DM without NAFLD diagnosis were excluded.

#### Intervention

We included trials that assessed the effect of lifestyle interventions, such as dietary changes and/or exercise interventions.

Dietary interventions were defined as any diet with calorie restriction, low fat, or low carbohydrate composition and intermittent feeding. Exercise was defined following Caspersen`s definition: “physical activity that is planned, structured, repetitive, and purposive in the sense that improvement or maintenance of one or more components of physical fitness is an objective” [17]. Physical fitness includes health-related components such as cardiorespiratory endurance, muscular endurance, muscular strength, body composition, and flexibility.

There were no restrictions of exercise dosage in terms of modality, intensity, frequency, or duration.

#### Comparator

We included studies that compared intervention with conventional, standard, or optimal treatment or no treatment, as defined by the authors of the trials. Studies that compared two types of exercise or two types of diet without a control group were excluded.

#### Outcomes

Once the article met the inclusion criteria, we evaluated studies assessing the next outcomes:

1. **Primary outcomes**. Quality of life (QoL) evaluated by different questionnaires as defined by the included studies. These were evaluated at the end of the follow-up.
2. **Secondary outcomes**. As secondary outcomes we considered IHL assessed by MRS, MRI, or US; serum liver enzymes concentrations, especially alanine aminotransferase (ALT) and aspartate aminotransferase (AST); changes in body weight, CRF evaluated with maximum oxygen consumption or peak oxygen consumption (VO_2_ ml/kg/min), and HOMA-IR as a parameter of glucose metabolism.

### Search methods for identification of studies

#### Electronic searches

To build the search strategy the following terms were considered: *Nonalcoholic fatty liver disease*, *NAFLD*, *liver steatosis*, *fatty liver*, *steatohepatitis*, *NASH*, *exercise*, *physical activity*, *aerobic exercise*, *aerobic*, *endurance exercise*, *endurance*, *resistance exercise*, *physical training*, *exercise training*, *sport*, *train*, *fitness*, *yoga*, *Pilates*, *flexibility*, *Tai Chi*, *diet*, *calorie restriction*, *diet therapy*, *lifestyle changes*, *lifestyle intervention*, *lifestyle modification*.

#### Databases

The Cochrane Central Register of Controlled Trials (CENTRAL) in the Cochrane Library (latest issue), EMBASE and PubMed. Searches included a cover from the inception date of each database until the day before submission. The strategies for each database are reported at the end of text (S2 File).

#### Searching for other sources

In order to identify articles that might have been missed in the electronic searches, we searched for primary studies in the following sources: systematic reviews about NAFLD and lifestyle changes, online trial registries such as ClinicalTrials.gov (clinicaltrials.gov) and WHO International Clinical Trial Registry Platform (www.who.int/ictrp) for ongoing or unpublished trials, Google Scholar and Microsoft Academic for cross-citation searches using each included study as the index reference and reference lists of included studies to identify additional trials for inclusion.

### Data collection and analysis

#### Selection of studies

Two authors (TF and MV) independently reviewed and selected studies in two stages: 1) title and abstract screening; 2) full-text screening based on the inclusion criteria described above. Discrepancies at either stage were discussed by both authors and a third author acted as an arbitrator (FB).

#### Data extraction and management

Using standardized forms, TF and MV independently extracted data from each included study. The collected data included: DOI, study design, participants characteristics (age, sex, weight, BMI, HOMA-IR, liver enzymes levels, intrahepatic triglycerides levels, NAFLD stage and comorbidities); geographic location, study eligibility criteria; details about the intervention and comparison, such as calorie restriction, diet composition and duration in dietary intervention; exercise modality, intensity, frequency and duration of exercise protocol; the outcomes assessed and the time they were measured; funding source of the study; and conflicts of interest disclosed by the investigators. Disagreements were solved through dialogue, and one arbiter (FB) made decisions in unresolved disagreements.

#### Assessment of risk of bias in included studies

Risk of bias in the included studies was assessed by two independent reviewers using the Cochrane Risk of Bias’s tool, which included the following items: random sequence generation, allocation concealment, blinding of participants and personnel, blinding of outcome assessment, incomplete outcome data, selective reporting, and other sources of bias. The risk of bias was classified as high, low or unclear, according to the methods described in Chapter 8 of the Cochrane Handbook for Systematic Reviews of Interventions [18]. Disagreements were discussed and solved through consensus.

#### Measures of treatment effect

Data was analyzed using Review Manager (Revman 5.3, 2014). To summarize the data, we used mean difference (MD) and standard deviation (SD) as most outcomes were expressed as continuous variables. Whenever these outcomes were measured using different scales, the treatment effect was expressed as a standardized mean difference (SMD) with 95% confidence interval. We used Cohen’s thresholds to interpret the clinical relevance of effect size results, in which < 0.2 corresponds to small effect size, ≥ 0.2 and < 0.5 corresponds to moderate effect size and ≥ 0.5 and <0.8 or ≥ 0.8 corresponds to a large effect size.

#### Dealing with missing data

We contacted twelve authors of included trials to request additional data. When the statistics were not provided, they were calculated using the Revman 5.3 (2014) Calculator, and formulas as mentioned in Luo et al. and Wan et al. [19, 20].

#### Assessment of heterogeneity

We assessed heterogeneity through the I^2^ test, as it does not depend inherently on the number of studies included. We presented the I^2^ to gauge the degree of heterogeneity of the sample. The following guidance was used to quantify heterogeneity using the I^2^ statistic: 25% indicates low heterogeneity, 50% indicates moderate heterogeneity and 75% indicates high heterogeneity [18].

#### Data synthesis

Because of the heterogeneity of interventions, considering diet, exercise, and the combination of both, we restricted our inclusion criteria to trials that only compared lifestyle changes to standard, optimal, and conventional care, or no intervention.

We presented a narrative synthesis of the data when it was insufficient to calculate an effect estimate or when studies were not comparable because of the heterogeneity of assessment methods, intervention, or control group. We described the studies in terms of direction and magnitude of their effects and exposed any precision measure available. Whenever data was shared among different studies, we conducted a formal quantitative synthesis (meta-analysis) using Excel and Revman 5.3 (2014). We applied a random effect model on the studies in which data heterogeneity was greater than 50%.

To include those studies with more than one intervention group, we combined them to undertake a single pair-wise comparison according to the formula by Cochrane’s handbook [18].

#### Subgroup analysis

We undertook the following subgroup analyses once the number of studies was sufficient:

- Type of intervention: a priori, we planned to compare studies that included only diet, only exercise, or both.
- Length of interventions: we compared studies by duration, considering the following groups: less or equal to eight weeks, more than eight weeks but less than 24 weeks and more than 24 weeks
- Supervision

#### Certainty of evidence

We assessed quality of evidence for included outcomes according to Grades of Recommendation, Assessment, Development and Evaluation (GRADE) methodology for risk of bias, inconsistency, indirectness, imprecision, and other considerations [21].

## Results

### Results of the search

**Fig 1:**
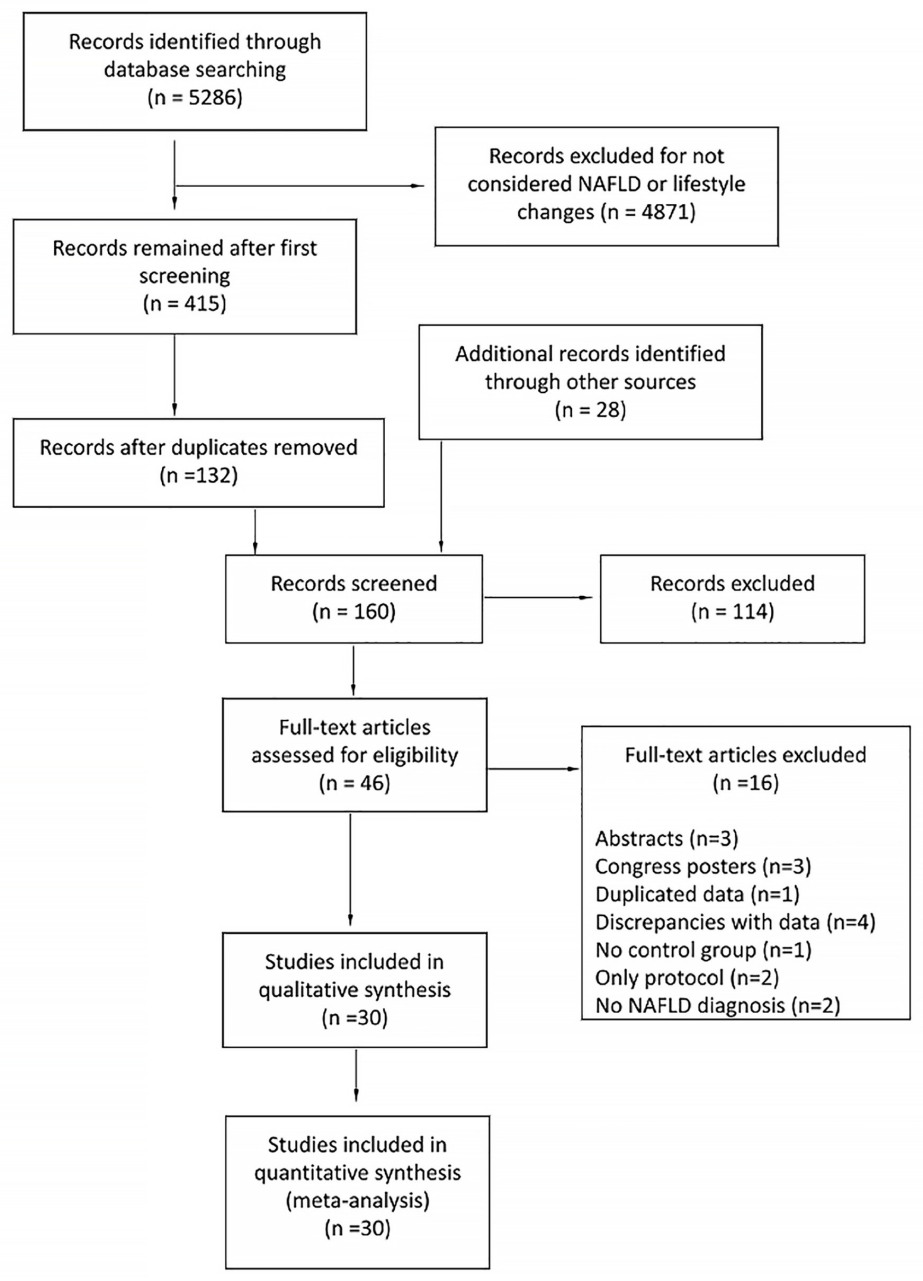
Flowchart of studies selection process.

A systematic literature search was conducted by TF and MV in Cochrane Central Register of Controlled Trials (CENTRAL) in the Cochrane Library (latest issue), EMBASE and PubMed from May 2020 to August 2020. 5286 studies were identified according to search strategies. Using *Collaboratron* tool by Epistemonikos, the two authors independently selected 132 relevant studies as reported in Fig 1. After scanning these studies and checking the cross-references, 28 more studies were found. From a total of 160 studies evaluated, 38 studies were not randomized controlled trials, 34 did not meet the inclusion criteria, four were in a different language, six were not found in databases, four were just registered protocols, eight considered the effectiveness of monitoring lifestyle changes but not the effect itself of the intervention, and 20 were duplicates.

From 46 studies selected to be fully analyzed, 16 [22–37] were excluded the reasons as mentioned in Fig 1.

#### Included studies

We included 30 studies that were all randomized controlled trials published between 2011 and 2020 performed mostly in Asia, Europe, and America (Table 1).

**Table 1:** Characteristics of the evaluated population.

Author, year	Location	n	M/F (n)	Baseline: Mean age (years)	Diagnosis method	Comorbidities
Abdelbasset, 2019	Egypt	32	19/13	INT: 54,4 ± 5,8 CON: 55,2 ± 4,3	Guidelines for NAFLD in the Asia-Pacific region	Type II diabetes, obesity type II or III
Abdelbasset, 2020	Egypt	32	17/14	INT: 54.9 ± 4.7 CON: 55.2 ± 4.3	Guidelines for NAFLD in the Asia-Pacific region	Type II diabetes, obesity type II or III
Al-Jiffri, 2013	Saudi Arabia	100	No details	non-specified	Elevated AST and/or ALT, liver biopsy	Type II diabetes, obesity
Arab, 2017	Iran	82	22/47	INT: 49,45 ±1,46 CON: 48,23 ±1,63	Liver sonography and laboratory test	Overweight or obesity
Cai, 2019	China	271	87/205	ADF: 35,50 ± 4,417, TRF: 33,56 ± 6,23, CON: 34,54 ± 6,96	Abdominal ultrasound with liver stiffness > 9.6 kpa	No
Cheng, 2017	China	115	26/89	INT: 59 (4,4) DIET: 60 (4,1) AED: 60 (3,5) CON: 60 (3,4)	H-MRI (liver fat>5%)	Impaired fasting insulin or glucose
Cuthbertson, 2015	England	69	No details	INT: 52 (46, 59), CON: 50 (46, 58)	Diagnosed clinically by a hepatologist	Obesity
Dong, 2016	China	280	Only males	INT: 56.68 ±5.33, CON: 57.94 ± 5.71	Guidelines for NAFLD in the Asia-Pacific region + Ultrasound	No
Eckard, 2013	United States	56	29/29	LFDE: 44 ± 11, MFDE: 55 ± 5, ME: 52 ± 10, CON: 51 ± 11	Liver biopsy	Hyperlipidemia, diabetes type II, prediabetes, hypertension
Ghetti, 2019	Brazil	44	21/19	INT: 48,3 CON: 50,6	Liver biopsy	Metabolic Syndrome
Hallsworth, 2011	United States	21	Only males	INT: 52 ± 13.3 CON: 62 ± 7.4	H-MRS (> 5% IHL and a score of less than -1.445 on the NAFLD fibrosis scoring system)	Type II diabetes
Hallsworth, 2015	United States	29	No details	INT: 54 ± 10 CON: 52 ± 12	H-MRS (>5% and a score of less than −1.455, Kleiner F3/4)	No
Houghton, 2016	United Kingdom	26	Only males	INT: 54 ± 12 CON: 51 ± 16	Liver biopsy	No
Johari, 2019	Malaysia	43	33/10	INT: 45.33 (10.77) CON: 52.60 (12.03)	Liver enzymes alterations	Dyslipidemia, Type II diabetes
Katsagoni, 2018	Greece	63	43/20	MDG: 44 (41–60), MLG: 48 (38–60), CON: 47 (42–60),	Ultrasound and liver enzymes alterations	Overweight or obesity
Marin-Alejandre, 2019	Spain	98	51/47	FLiO: 49.2 (8.9) CON: 51.1 (9.8)	Ultrasonography	Overweight or obese
Nikroo, 2017	Iran	25	Only males	INT: 38.67 ± 7.36, CON: 35.64 ± 9.22	Ultrasonography and ALT alterations	No
Nourian, 2020	Iran	82	22/47	INT: 49.45 ± 1.46 CON: 48.23 ± 1.63	Sonography, and liver enzymes alterations	Overweight, obesity, dyslipidemia
Promrat, 2010	United States	31	22/9	INT: 48,9 ± 10,9	Elevated aminotransferases (AST and/or ALT) and liver biopsy	Metabolic Syndrome, diabetes
Pugh, 2014	England	31	30/24	INT: 48 ± 2 CON: 47 ± 2	H-MRS. Intrahepatocellular lipid > 5,5%	Obesity
Razavi, 2015	Iran	60	30/30	INT: 39.7 ± 7.3 CON: 42.8 ± 10.6	Ultrasonography scan and increased levels of serum ALT levels	Obesity or overweight
Rezende, 2015	Brazil	44	Only females	INT: 56.2 ± 7.8 CON: 54.5 ± 8.9	Liver biopsy	Hypertension, dyslipidemia, diabetes mellitus, post menopause
Shamsoddini, 2015	Iran	30	Only males	AE: 39.7 ± 6.3 RES: 45.9 ± 7.3 CON: 45.8 ± 7.3	Ultrasonography. Hepatic triglyceride content greater than 5%	No
Shojaee-Moradie, 2016	United Kingdom	27	Only males	INT: 52.4 ± 2.2 CON: 52.8 ± 3.0	Serum transaminases, hepatic steatosis on ultrasound or by liver biopsy	No
Sullivan, 2012	United States	33	5/13	INT: 48.6 ± 2.2 CON: 47.5 ± 3.1	IHTG >10%	Obesity
Sun, 2012	China	1087	646/359	INT: 37.9 ± 12.3 CON: 36.4 ± 17.2	Ultrasound	Diabetes and Metabolic Syndrome
Takahashi, 2015	Japan	64	17/42	INT: 56.7 ± 12.0 CON: 52.9 ± 15.4	Asia-Pacific Working Party guidelines for NAFLD	T2DM, Hypertension
Yao, 2018	China	103	36/55	AE: 61.28 ± 7.52 RES: 55.80 ± 12.29 CON: 58.06 ± 9.79	2010 guidelines for diagnosis and treatment of NAFLD	No
Zelber-Sagi, 2014	Israel	82	34/30	INT: 46.32 ± 10.32 CON: 46.64 ± 11.4	Ultrasonography. HRI> 1.5	No
Zhang, 2016	China	220	71/149	MOD: 54.4 (7.4) VIG: 53.2 (7.1) CON: 54.0 (6.8)	H-MRS (IHTG content ≥5%)	Central Obesity

AE: aerobic exercise, ADE: Aerobic exercise + diet, ADF: Alternate-day fasting, CON: control, INT: intervention, LFDE: Low fat diet + moderate exercise, MDG: Mediterranean diet group, ME: Moderate exercise, MFDE: moderate fat with low-processed carbohydrates + moderate exercise, MLG: Mediterranean lifestyle group, MOD: moderate exercise, RES: resistance exercise, TRF: Time restricted feeding, VIG: vigorous exercise.

In terms of the interventions, 16 studies considered exercise alone [38–53]. 6 considered diet alone [54–59] and eight considered diet combined with exercise [24, 60–66]. Accordingly, eleven, three, and only one of the studies were supervised by a professional.

Control group varied among studies. In the exercise alone studies, six considered conventional or standard treatment without more details, three considered education or advise about NAFLD, exercise, and healthy eating; one considered stretching as recommended by the ACSM’s guidelines, four studies considered maintaining normal physical activity and two studies considered diet as the base treatment for both groups with the addition of exercise in the intervention group. In diet alone studies, three had control groups with different diets; two included usual care without more details; and one only considered nutritional orientation. Finally, in the studies with combined exercise and diet intervention; two studies had a control group without intervention, two considered standard care; and four studies considered education or advice (Tables 2–4).

**Table 2:** Trials evaluating exercise alone in NAFLD outcomes.

Author, year	Type of intervention	Supervision	Duration	Intervention group protocol	Control group protocol	Outcomes	Changes post intervention
Abdelbasset, 2019	Exercise	No	8 weeks	5-minute warm-up. 3 sets of 4-min cycling sessions at 80–85% VO2max with 2-minute interval at 50% of the VO2max between sets. 5 minutes of cool-down.	Medical treatment	IHTG, visceral lipids, CLDQ, cardiorespiratory fitness, plasma glucose, ALT	↓ BMI*☨
							↓ IHTG*☨
							↓ TC, TG, HDL,
							LDL*☨
							↓ HOMA-IR*☨
							↓ ALT*☨
							↑ VO2*☨
							↑ QoL*☨
Abdelbasset, 2020	Exercise	No	8 weeks	5 minutes warm-up. Cycling with continuous intensity at 60–70% of MHR. 5 minutes cooling-down.	Medical treatment	IHTG, visceral lipids, lipid profile, insulin sensitivity, HbA1c, ALT	↓ BMI*
							↓ TG, TC, HDL,
							LDL*
							↓ IHTG*
							↓ ALT*
							↓ HOMA-IR, HbA1c*
Cuthbertson, 2015	Exercise	Yes	16 weeks	Aerobic progressive exercise (30% HRR) progressing weekly based on HR responses (5/week 45 min at 60% HRR by week 12).	Advice about the health benefits of exercise	Weight, VAT, SAT, IHCL, IMCL, plasma glucose, cardiorespiratory fitness, TC, liver enzymes, HOMA, blood pressure	↓Weight*☨
							↓BMI *☨
							↓IHCL*☨
							↑VO2 peak*☨
							↓ALT, AST, GGT *
							↓TG, TC, LDL
							↓ blood pressure*
							⏤Fasting glucose
							↓VAT*, SAT*☨, abdominal fat *☨
Hallsworth, 2011	Exercise	Yes	8 weeks	10 min warm-up (60% maximum heart rate on a cycle ergometer). Resistance exercise done as a circuit (8 exercise) and cool- down. Initially, 2 circuits using 50% of RM progressing to 3 circuits, 70% of their one repetition maximum by week 7	Normal treatment	Weight, body composition, IHL, subcutaneous and visceral fat, glucose, insulin, HOMA-IR, ALT, TC, HbA1c	⏤BMI
							⏤Weight
							↓IHL*
							⏤VAT, SAT
							⏤Fasting glucose, fasting insulin
							↓HOMA-IR*
							⏤Blood lipids
							⏤ALT
							↑Fat oxidation in submaximal test
							⏤HbA1c
Hallsworth, 2015	Exercise	No	12 weeks	5-min warm up progressing on Borg perceived exertion of 9 to 13. 5 intervals of cycling at an RPE of 16–17 (‘very hard’) x 3-min recovery periods. 3-min cool down after the last interval.	Normal treatment	Weight, body composition, IHL, blood glucose, plasma insulin, HOMA 2, liver enzymes, lipid profile, HbA1c, cardiac function	↓Weight*
							↓BMI*
							⏤VAT
							↓IHL*☨
							↓Liver enzymes*☨
							⏤Fasting glucose and insulin
				2 min-long intervals at first week adding 10 s per week. By week 12, interval was 3 min 50 s long. Recovery periods included 90 s of passive recovery, 60 s of light band resisted upper body exercise and 15 s each to transition off and on the ergometer.			
							⏤HbA1c
							⏤TC
							↑Diastolic filling rate*
Houghton, 2016	Exercise	Yes	12 weeks	5-minute warm-up. 3 intervals on a fixed bike for 2 min x 1-minute rest in-between. Intensity: Borg RPE (6–20 points) with bike intervals corresponding to a RPE of 16 to 18 (very hard). Resistance exercise circuit that comprised 5 exercises. Intensity: weight for each resistance exercise based on a rating of perceived exertion of 14 to 16 (hard)	Standard care	HTGC, body composition, fasted blood samples, inflammatory and fibrosis markers	↓HTG*☨
							↓TG*☨
							↓VAT*☨
							↓GGT*☨
							—BMI
							—Weight
							—SAT
							—Fasting glucose and insulin
							—HOMA-IR, HbA1c
							— inflammatory and fibrosis biomarkers
Nikroo, 2017	Exercise	Yes	8 weeks	Same diet as control group with aerobic training followed ACSM’s recommendations. 55–60% of HRR, 15 minutes of warm-up, 10–25 minutes of aerobic exercise, 10 minutes of cool down.	Indivualized diet. 500 kcal of energy less than estimated daily energy requirement. CHO 60%, fat 25%, protein 15%.	Weight, BMI, WC, WHR, body fat, arterial blood pressure, ALT, AST, TC, LDL, HDL, TG Insulin, HOMA-IR, VO2 peak, FBS	↓Weight*☨
							↓BMI*
							↓WHR*☨
							↓blood pressure*
							↓AST, ALT*☨
							↓TG*☨
							↓Insulin*
							↓HOMA-IR*
							↑VO2*☨
Pugh, 2014	Exercise	Yes	12 weeks	Treadmill and cycle-ergometer based exercise. 1–4 week: 30% HRR. 5–8 week: increased to 45% HRR. 9–12 week: 45% HRR increasing to 45 min. From week 12. No dietary intervention.	Standard care	BMI, anthropometrics and body composition, arterial blood pressure, cardiorespiratory fitness, liver enzymes, lipid profile, glucose, insulin, HOMA-IR, liver fat, cardiac parameters	—BMI
							—Weight
							↓WC*☨
							—Blood pressure
							↑VO2*☨
							↓Fasting glucose*☨
							—Insulin
							—Liver enzymes
							—Lipid profile
							—Liver fat
							↓FMD*☨
Rezende, 2015	Exercise	Yes	24 weeks	Same diet as control group plus 5-minute warm up followed by 30 to 50 minutes of treadmill aerobic exercise and 5 minutes of cooling down	Standardized diet. Energy deficit of 500 kcal/d. 35% protein, 25% lipids, and 40% carbohydrates	Weight, BMI, body composition, glucose, HbA1c, lipid profile, liver enzymes, ferritin, HOMA-IR, liver stiffness, cardiorespiratory fitness	↓BMI*☨
							↓WC*☨
							—Liver enzymes
							—Glycemic profile
							—Fibrosis stages
							↑VO2*☨
Shamsoddini, 2015	Exercise	Yes	8 weeks	AER: 10 minutes warm-up. 2 x 15 minutes running on treadmill at 60% MHR in the first week and increased to 2 x 15 min running at 75% MHR per week at final.	No exercise program- daily physical activity	Height, weight, BMI, body fat, fat mass, waist and hip circumference, subcutaneous body fat, liver enzymes	↓BMI*☨ (only resistance)
							↓Weight
							↓ALT, AST*☨
							↓Hepatic fat*☨
							↓HOMA-IR*☨
							(only aerobic)
				RES: 5-minute warm-up. Circuit of 7 resistance exercise. 5-minute cool down. 1–2 week: 2 x 50% of RM x 10 repetitions, 3–4 week: progressing to 2 x 60% of 1RM x 10 repetitions. 5–6 weeks, 3 x 60% of their 1RM x 10 7–8 weeks: 3 x 70% of their 1RM x 10 repetitions. 90 s rest			
Shojaee-Moradie, 2016	Exercise	Yes	16 weeks	Exercise at moderate intensity (40%–60% heart rate reserve), based aerobic plus resistance exercise, or outdoor aerobic activities and resistance exercise.	Conventional lifestyles advise	Weight, BMI, waist circumference, cardiorespiratory fitness fasting glucose and insulin, HOMA2%S, adipose tissue-IR, lipid profile, liver enzymes, IHTG, arterial blood pressure, body composition, Framingham risk, lipid kinetics	↓BMI*☨
							↓Weight*☨
							↓Liver enzymes*☨
							↓IHCL*☨
							↓Fasting glucose, insulin*
							↓Blood pressure *☨
							↓Framingham score*☨
							—TG, TC, HDL
							↓LDL*
							↑VLDL1-TG, VLDL1-ApoB kinetics*
Sullivan, 2012	Exercise	Yes	16 weeks	Aerobic exercise at 45%-55% of their V̇O2 peak. initiating by walking on a treadmill for 15–30 minutes at a HR equivalent to 45–55% of their pretraining V̇O2 peak, and progressively increased until 30–60 min of moderate intensity exercise, 5 times a week.	Current activities of daily living	BMI, body mass, body fat mass, fat free mass, cardiorespiratory fitness, lipid profile, ALT, IHTG, lipid kinetics	↑VO2 peak*
							—Weight
							—BMI
							↓IHTG*☨
							—Lipid profile
							↓ALT*☨
							—Lipid kinetics
Takahashi, 2015	Exercise	No	24 weeks	Resistance exercise consisted of push-up and squats. 3 sets of 10 push-ups and 3 sets of 10 squats with a 1-min interval between each set over a period of 20–30 min.	Education about dietary restrictions + regular physical activities	Weight, BMI, body composition, liver enzymes, gamma-GTP, lipid profile, ferritin, blood glucose, insulin, HOMA-IR, HbA1c, hepatic steatosis	↓Weight*☨
							↓BMI*☨
							↓ALT*☨
							↓HOMA-IR*
							↓Hepatic steatosis
							grade*☨
							—Lipid profile
							—Glycemic
							profile
Yao, 2018	Exercise	Yes	22 weeks	AER: warming up (5 min), training (50 min) and relaxing (5 min). Progressed from 40 min/day at 45%-55% MHR intensity (within first 2 weeks) and increased to 60 min/day at 60%-70% MHR.	Maintain daily physical activities and education about NAFLD and treatment	Weight, BMI, WC, hip circumference, fasting glucose and insulin, blood lipids, liver enzymes	—BMI
							↓TG*☨
							↓ALT*☨
							—Fasting glucose and insulin
							↓HDL*☨
				RES: warm up (5 min joint movement), training (50 min), and relaxing (5 min). 3 series x 8 repetitions, 30%-40% of 1RM for 40 min/day (within first 2 weeks) gradually move to 3 series x 10 repetitions, 60%-70% 1RM for 60 min/ day, with one minute of recovery between series			
Zelber-Sagi, 2014	Exercise	No	13 weeks	8 resistance exercise. 8–12 repetitions x 3 sets x 1–2 min rest between sets. Load was gradually increased by 2%-10% in the following training sessions, according to the individual ability of the patient.	Home stretching routine followed the ACSM’s guidelines	HRI score, weight, BMI, body fat, liver enzymes, serum glucose, insulin, glycosylated hemoglobin, cholesterol. TG	↓HRI score *☨
							↓Weight*☨
							↓BMI*☨
							↓Body fat*☨
							—Liver enzymes
							↓TC*☨
							—Glucose, insulin, HbA1c
Zhang, 2016	Exercise	Yes	52 weeks	VIG: Treadmill increasing intensity from 65 to 80% of MHR MOD: briskly walk, 120 steps/min. HR from 45% to 55% of MHR	Education sessions and encourage to not change daily physical activity	Physical activity, total energy intake, fat intake, HR, blood pressure, serum TG, serum total cholesterol, HDL, LDL, visceral fat, subcutaneous fat, body fat, IHTG, weight, waist c.	↓IHTG*☨
							↓Weight, WC*☨
							↓Body fat mass *☨ (compared to CON and MOD)
							↓Blood pressure*☨
							—Lipid profile
							—Fasting glucose
							—ALT, GGT
							↑AST in VIG

* Statistically significant within group,

^☨^ statistically significant compared to control group,

↓ decreased, ↑ increased, — no change. Abbreviations: AER: aerobic, ACSM: American college of sports medicine, ALT: alanine aminotransferase, AST: aspartate aminotransferase, BMI: body mass index, CHO: carbohydrates, FBS: fasting blood sugar, GGT: gamma-glutamyl transferase, HbA1c: glycated hemoglobin, HDL: high-density lipoprotein, HOMA-IR: homeostatic model assessment for insulin resistance, HRI: HRR: heart rate reserve, HTGC: hepatic triglyceride content, IHCL: intrahepatocellular lipids, IHTG: intrahepatic triglycerides, IMCL: intramyocellular lipid, LDL: low- density lipoprotein, MHR: maximum heart rate, QoL: quality of life, RES: resistance, RM: maximum repetition, SAT: subcutaneous adipose tissue, TC: total cholesterol, TG: triglycerides, VAT: visceral adipose tissue, VLDL: very low density lipoprotein, VO_2_ max: maximum oxygen consumption, WC: waist circumference, WHR: waist to hip ratio.

**Table 3:** Trials evaluating diet alone in NAFLD outcomes.

Author, year	Type of intervention	Supervision	Duration	Intervention group protocol	Control group protocol	Outcomes	Changes post intervention
Cai, 2019	Diet	No	12 weeks	ADF: 25% of their baseline energy needs on the fast day (24 h). Ad libitum at home on the feed day (24 h).	80% of energy needs with no other recommendations	Weight, waist circumference, liver stiffness, lipid profile, glucose	↓Weight*
							—BMI
							↓Fat mass*
							—WC
				TRF: Meal within an 8-h window and asked to refrain from consumption of all food or beverages that included energy for the remaining 16 h.			
							↓Cholesterol HDL, LDL, TG☨
							—Glucose
							—Liver stiffness
Ghetti, 2019	Diet	No	13 weeks	Diet: Individualized diet plus nutritional orientation. Overweight or obese outpatients received a hypocaloric diet (500 to 750 cal/day less).	Only nutritional orientation	Liver biopsy, liver enzymes, lipid profile, fasting glucose and insulin, anthropometric measures, BMI, fecal microbiota	↓Weight*
							↓BMI*☨
							↓Glucose*
							↓Insulin☨
							↓HOMA-IR*☨
							↓TC, TAG*
							—HDL, LDL
							↓AST*☨, ALT*, GGT*☨
Johari, 2019	Diet	Yes	8 weeks	Fasting day: restrict 70% of the calorie requirement per day. Calorie-deficient meals were consumed between 2–8 pm. non-fasting day: ad libitum, normal diet. Self-selected diet plans.	Usual habitual diet	Weight, BMI, liver enzymes, lipid profile, fasting blood glucose, liver steatosis, SWE (kPa)	↓Weight*☨
							↓BMI*☨
							—Lipid profile
							↓ALT*☨, AST*
							↓Liver steatosis grading*☨
							↓Blood glucose*
Marin-Alejandre, 2019	Diet	No	26 weeks	FLiO: higher meal frequency (7 meals/day). 40%–45% CHO (low glycemic index), 25% proteins (vegetable sources), and 30%–35% lipids	AHA: 3–5 meals/day. 50%–55% from CHO, 15% from proteins, and 30% from lipids.	Weight, BMI, body composition, lipid profile, fasting glucose, insulin, HOMA-IR, leptin, adiponectin, C-reactive protein, liver enzymes, hepatic volume, liver fat, liver stiffness, dietary intake, physical activity	↓Weight*
							— TC, HDL,
							LDL
							↓TG*
							↓ HOMA-IR, fasting
							glucose, insulin*
							↓ ALT, GGT
							↓ Liver fat*
							— Liver stiffness
							↑HBM variables*☨
							↓AST, ALT*☨
							↓Fatty liver grade*☨
Nourian, 2020	Diet	No	9 weeks	Lesson plan based on HBM. Education about increasing the intake of fruits, vegetables, complex carbohydrate, low dairy fat, healthy fat, white meat, and fish and avoid the intake of unhealthy fats and refined carbohydrate	Usual care	HBM variables, AST, ALT, Fatty liver grade	
Razavi, 2015	Diet	No	8 weeks	DASH: 52–55% CHO, 16–18% proteins and 30% total fats. Rich in fruits, vegetables, whole grains, and low-fat dairy products and low in saturated fats, cholesterol, refined grains, and sweets.	Calorie-restricted diet (350–700 kcal less than energy requirement for each person; 350 kcal for patients with the BMI in the range of 25–27.5 kg/m2; 500 kcal for those with the BMI in the range of 27.5–31 kg/m2; and 700 kcal for those with the BMI >31 kg/m2) to avoid ethical problems	Grade of fatty liver, waist circumference, hip circumference, liver enzymes, PFG, insulin, HOMA-IR, QUICKI, lipid profile	↓Weight*☨
							↓BMI*☨
							↓NAFLD grade*
							Change from NASH to NAFLD 80% of patients
							↓Liver enzymes*☨
							↓HOMA-
							IR*☨
							↓Insulin*☨
							↓WC*☨
							↓Lipid profile*☨
							↑QUICKI*☨

* Statistically significant within group,

^☨^ statistically significant compared to control group,

↓ decreased, ↑ increased, — no change. Abbreviations: ADF: alternate-day fasting, ALT: alanine transferase, AST: aspartate transferase, BMI: body mass index, GGT: gamma glutamyl transferase, HBM: health belief model, HDL: high density lipoprotein, HOMA-IR: homeostatic model assessment of insulin resistance, LDL: low density lipoprotein, QUICKI: quantitative insulin sensitivity check index, SWE: shear wave elastography, TAG: triacyl glyceride, TC: total cholesterol, TG: triglycerides, TRF: time restricted feeding, WC: waist circumference.

**Table 4:** Trials evaluating combination of diet and exercise in NAFLD parameters.

Author, year	Type of intervention	Supervision	Duration	Intervention group protocol	Control group protocol	Outcomes	Changes post intervention
Al-Jiffri, 2013	Exercise + Diet	No	13 weeks	EX: 5-minute warm-up phase on the treadmill. Endurance training session for 30 minutes. 5-minute recovery	No intervention	liver enzymes, HOMA-IR, insulin	↓BMI*☨
							↓ALP, ALT, AST,
							GGT*☨
							↓HOMA-IR*☨
				D: low calorie diet (1200 kcal): 15% as protein, 30 to 35% as fat and 50 to 55% as CHO			
Arab, 2017	Exercise + Diet	No	9 weeks	Eight lifestyle sessions about healthy eating and regular physical activity for 5 days/week	Usual care	Weight, BMI, other body composition measures	↓Weight*
							↓BMI*☨
							↓Abdominal circumference*
							↓Lean body mass*
							↓Percent of body fat*
Cheng, 2017	Exercise + Diet	Yes	37 weeks	AER: Progressive aerobic training program. 5 min warm-up, 30–60 minutes session, 5 minutes cool-down. 65 to 70% of VO2max.	No intervention	HFC, HbA1c, insulin sensitivity, body composition, total cholesterol, liver enzymes	↓HFC*☨
							↓Weight (only in AED group)
							↓FM☨
							↓fat% ☨
							↓HbA1c (only AED group)
				D: 30–40%of the total daily energy intake. 37–40% CHO, 9–13 gr as fiber, 35–37% fat, 25–27% protein. Maintain personal physical activities			
				AED: AER + D protocols.			
Dong, 2016	Exercise + Diet	Yes	104 weeks	D: Negative calorie balance of (25–30 c/kg/d) for overweight and obese. Neutral calorie balance (30–35 c/kg/d) for normal BMI. 20–30% fat, 15–20% protein, 50–60% CHO	Lifestyle counseling	Weight, liver enzymes, IHL content, lipid profile, fasting plasma glucose	↓Weight*
							—BMI
							↓NAFLD fibrosis score*
							↓ALT*, —AST, —GGT
							↓TC*, —TG,
							HDL*, LDL*
				EX: Favorite aerobic exercise or activity, moderate intensity (60–80% of heart rate) to vigorous (>80%).			
Eckard, 2013	Exercise + Diet	Yes	26 weeks	D: Nutrition prescription based on individualized calorie needs subtracting 500 kcal/day and macronutrient distribution	Standard care	Liver biopsy (NAS), body composition, lipid profile, liver enzymes, HbA1c, glucose, insulin	—Weight
							—Fat mass %
							↓NAS*
							↓ALT, AST*
							—Fasting glucose
							—Insulin
				EX: Education about exercise based on ACSM’s recommendations.			
Katsagoni, 2018	Exercise + Diet	Self-monitoring	26 weeks	Energy restriction regimen. 45% CHO, 20% protein, 35% lipids. 1500 kcal women, 1800 kcal men.	Energy restriction regimen. 45% CHO, 20% protein, 35% lipids. 1500 kcal women, 1800 kcal men + general written dietary guidelines for a healthy lifestyle.	Weight, BMI, WC, ALT, GGT, Liver stiffness, NAFLD fibrosis score, glucose, insulin, HOMA-IR, lipid profile	↓Weight*☨
							↓BMI*☨
							↓Liver stiffness*☨
							↓ALT only in MLG group*
				MDG: Seven 60-min small group sessions about improving diet quality and energy restriction held every 2 weeks for the first 2 months and every month for the next 4 months.			
				MLG: MDG + enhancing activity through a moderate–vigorous intensity physical activity program as well as for optimal sleep duration and mid-day rest.			
							—HOMA-IR
							↓LDL only MDG group*
							—blood pressure
							↑Adherence to intervention*
Promrat, 2010	Exercise + Diet	No	48 weeks	Weight loss intervention based on different strategies used successfully in other trials (diet, exercise, and behavior changes)	Small group sessions providing basic education about NASH, healthy eating, physical activity, and weight control.	Weight, BMI, ALT, AST, TC, LDL, HDL, TG, glucose, HbA1c, insulin, HOMA	↓Weight*☨
							↓WC*☨
							↓NAS*☨
							↓ALT*☨
							— HOMA, insulin, HbA1c
Sun, 2012	Exercise + Diet	No	12 weeks	Diet: 30% fat, 15% proteins and 55% carbohydrates, including 5% sugar. Energy intake: 25–30 kcal/kg. EX: walking, jogging, stair climbing and physical exercise, 23 (METs)•h/week (physical activity) + 4 METs•h/week (exercise).	Basic education about NAFLD and principles of healthy eating, physical activity, and weight control	Weight, BMI, WC, ALT, AST, GGT, TC, TG, fasting glucose, HOMA-IR, visceral fat area	↓Weight*☨
							↓BMI *☨
							↓WC*☨
							↓ ALT*☨
							↓ AST, GGT*
							— TG, TC, fasting glucose
							↓HOMA-IR*☨
							↓VFA*☨

* Statistically significant within group,

^☨^ statistically significant compared to control group,

↓ decreased, ↑ increased, — no change. Abbreviations: ACSM: American college of sports medicine, AED: aerobic exercise + diet, AER: aerobic, ALP: alkaline phosphatase, ALT: alanine transferase, AST: aspartate transferase, BMI: body mass index, CHO: carbohydrates, D: diet, EX: exercise, FM: fat mass, GGT: gamma glutamyl transferase, HbA1c: glycated hemoglobin, HDL: high density lipoprotein, HFC: hepatic fat content, MDG: Mediterranean diet group, MET: metabolic equivalent of task MLG: Mediterranean lifestyle group, TC: total cholesterol, TG: triglycerides, VFA: visceral fat area,VO_2_ max: maximum oxygen consumption, WC: waist circumference.

Twenty studies had two-arm interventions, while eight had three-arm interventions and only two studies had four-arm interventions.

Studies included 3280 participants in total, in which 37.6% were females (n = 1235). Three studies did not detail the gender of their participants, six studies only considered males as participants and one study had only females as participants. Ages ranged from 33 to 61 years old. Twenty-eight studies considered NAFLD patients confirmed by biopsy, H-MRI, H-MRS or ultrasound, whereas four papers included patients with NASH confirmed liver biopsy or ultrasound. Comorbidities reported were metabolic syndrome, obesity, type II diabetes, and hypertension.

Of the exercise studies, eight considered aerobic training, three consisted of a resistance training protocol, three combined aerobic and resistance training, and two studies had two exercise arm-interventions, in which one was aerobic, and the other was resistance exercise. In studies where intervention was diet plus exercise, seven studies included aerobic training, and only one included concurrent training. Training intensities varied from moderate to high intensity. Low intensity activities were only considered as the control (Tables 2–4).

Dietary intervention considered calorie restriction as the main diet protocol. Some studies reduced 500 kcal, others decreased 30–40% of the calorie intake, and others undertook a reduction of calories depending on the patient’s body mass index. There were two studies in which participants underwent an intermittent fasting protocol. One of them considered 25% of the baseline energy needs in the fasting day and the other used 30% of the calorie requirement per day. Two studies only gave advice on healthy food and four of them established diets such as the Mediterranean Diet, DASH diet (Dietary Approaches to Stop Hypertension) and FLiO diets (Fatty liver in Obesity created by Zulet MA., Abete I. and Cantero I. from Centro de Investigación en Nutrición, Facultad de Farmacia y Nutrición de la Universidad de Navarra) as intervention.

Time of the studies ranged from two months to six months and three studies had a longer duration (between 47 to 104 weeks) Table 2.

#### Risk of bias in the included studies

The overall risk of bias in each included study is presented in the S1 Table. Almost all studies had low risk of bias due to the randomization process, incomplete outcome data and selective reporting. Risk of bias was uncertain or high in the blinding of participants, personnel, and outcome assessment items due to poor reporting of this information or because participants or personnel were not blinded.

### Effects of lifestyle interventions in NAFLD

Meta-analysis was not feasible in the included outcomes because studies assessing diet alone were heterogeneous, not only in the intervention group but in the control group as well.

#### Primary outcomes

*Quality of life*. Meta-analysis was not feasible because only two studies included an assessed QoL using the Chronic Liver Disease Questionnaire (CLDQ) and SF-36 [39, 44].

Abdelbasset et al. [39] established that participants reported positive effects in all CLDQ’s domains after high-intensity aerobic exercise, showing a significant difference compared to no exercise. Nikroo et al. [44] concluded that after 12 weeks of aerobic exercise plus diet (intervention group), as well as diet alone (control), SF-36 was improved in patients with NASH, with no significant difference between both interventions (very low-quality evidence), Table 5.

**Table 5:** Summary of finding by GRADE quality assessment for exercise intervention.

Certainty assessment							№ of patients		Effect		Certainty	Importance
№ of studies	Study design	Risk of bias	Inconsistency	Indirectness	Imprecision	Other considerations	exercise alone	conventional treatment	Relative (95% CI)	Absolute (95% CI)		
**Body Weight**												
16	randomized trials	serious^a^	serious^b^	not serious	not serious	none	351	222	-	MD **2.64 Kg lower** (5.18 lower to 0.09 lower)	⨁⨁◯◯ Low	IMPORTANT
**Cardiorespiratory fitness**												
7	randomized trials	serious^a^	serious^c^	not serious	not serious	none	127	94	-	MD **6.18 ml/kg/min higher** (3.31 higher to 9.04 higher)	⨁⨁◯◯ Low	IMPORTANT
**Intrahepatic lipids**												
9	randomized trials	serious^a^	not serious	not serious	not serious	none	146	121	-	MD **1.95% lower** (3.27 lower to 0.63 lower)	⨁⨁⨁◯ Moderate	CRITICAL
**Alanine transferase**												
19	randomized trials	serious^a^	serious^b^	not serious	not serious	none	484	344	-	MD **2.97 U/L lower** (5.54 lower to 0.21 higher)	⨁⨁◯◯ Low	IMPORTANT
**Aspartate transferase**												
14	randomized trials	serious^a^	not serious	not serious	not serious	none	380	272	-	MD **0.84 U/L lower** (2.07 lower to 0.38 higher)	⨁⨁⨁◯ Moderate	IMPORTANT
**Homeostatic model assessment for insulin resistance**												
10	randomised trials	serious^a^	not serious	not serious	not serious	none	228	163	-	MD **0.46 lower** (0.8 lower to 0.2 lower)	⨁⨁⨁◯ Moderate	IMPORTANT

**CI**: confidence interval; **MD**: mean difference;

Explanations:

^a^. Unclear or high risk of bias,

^b^. Substantial heterogeneity with a I^2^ = 61%,

^c^. Substantial heterogeneity with a I^2^ = 87%.

#### Secondary outcomes

*Analysis by type of intervention*. *Weight*. Fifteen studies evaluated the effect of exercise alone, five included diet alone and five studies considered both interventions together in weight loss (Table 2).

**Fig 2:**
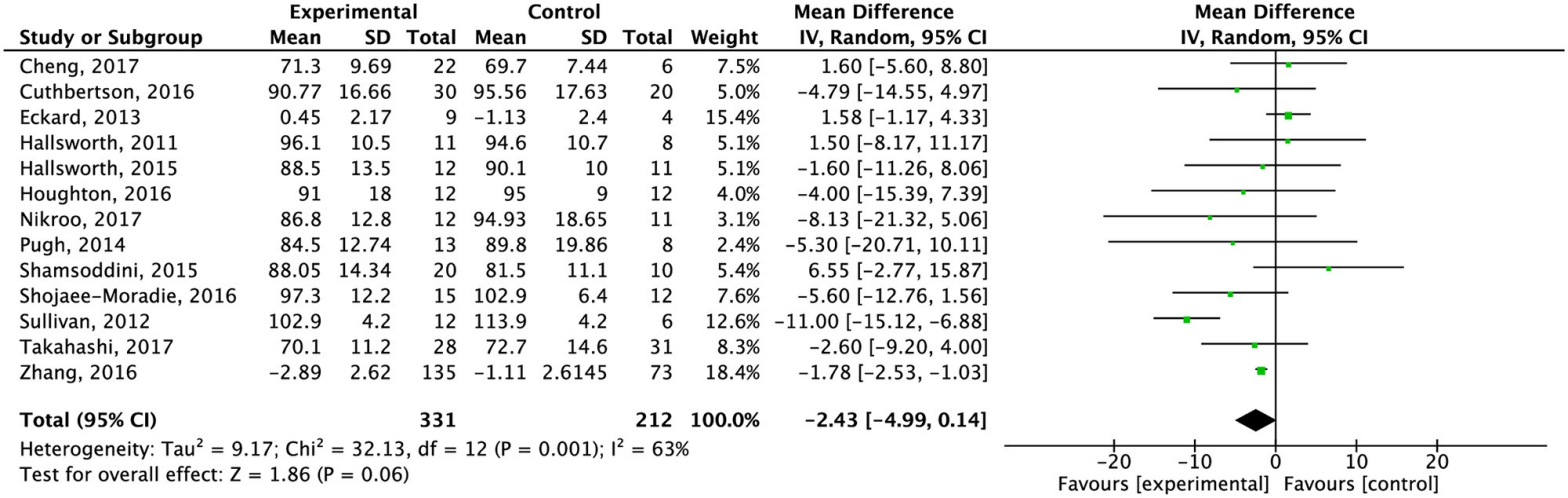
Effect of exercise alone compared to the control group in body weight.

Whenever studies were grouped by intervention type, exercise alone did not reduce weight significantly compared to the control group (high quality of evidence; Table 5) (Fig 2). After doing the analysis considering the type of control group, exercise significantly reduced weight compared to normal physical activity and education or advice about healthy lifestyle. No significant difference was seen compared to medical treatment (S1 Fig).

Of the five studies assessing diet alone, only two studies showed significant differences compared to their control group. Razavi et al. [59] assessed the effect of the DASH diet against the control diet with calorie restriction and 52–55% carbohydrates, 16–18% proteins and 30% total fat, and showed a reduction of -3.8 ± 2.2 kg, compared to -2.3 ± 1.7 kg. Johari et al. [55] assessed the effect of alternate-day calorie restriction compared to the usual habitual diet and showed a mean difference of 3.06 (1.14–4.63) kg.

**Fig 3:**
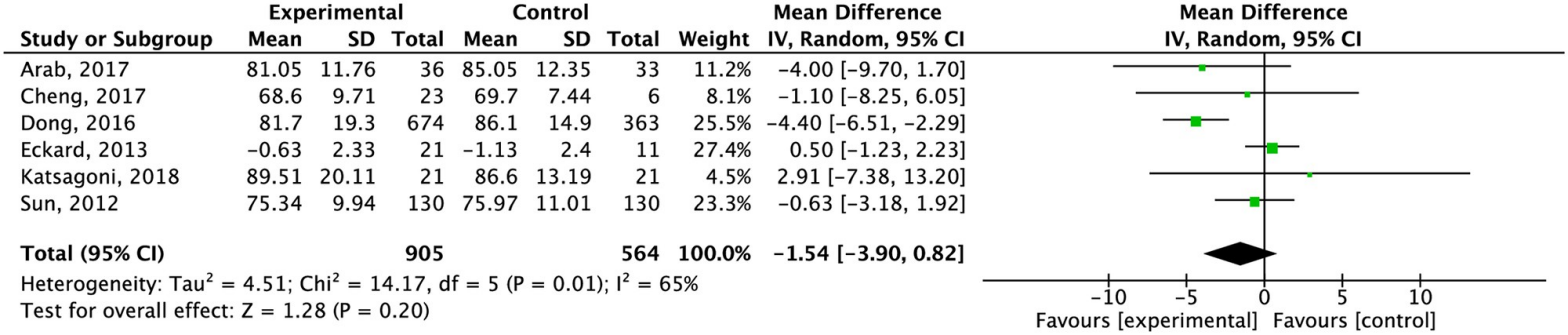
Effect of exercise plus diet compared to the control group in body weight.

The combination of exercise and diet did not significantly reduce weight compared to the control (-1.54 CI 95% -3.9, 0.83: low quality of evidence) (Table 6) (Fig 3). Compared to education about a healthy lifestyle or standard treatment separately, diet combined with exercise significantly reduced weight compared to education (-2.82 kg CI 95% -4.42, -1.21) (S2 Fig).

**Table 6:** Summary of finding by GRADE quality assessment for exercise plus diet intervention.

Certainty assessment							№ of patients		Effect		Certainty	Importance
№ of studies	Study design	Risk of bias	Inconsistency	Indirectness	Imprecision	Other considerations	exercise combined with diet	conventional treatment	Relative (95% CI)	Absolute (95% CI)		
**Body weight**												
6	randomized trials	serious^a^	serious^b^	not serious	not serious	none	905	564	-	MD **1.54 kg lower** (3.9 lower to 0.82 higher)	⨁⨁◯◯ Low	IMPORTANT
**Alanine transferase**												
6	randomized trials	serious^a^	very serious^c^	not serious	serious^c^	none	919	581	-	MD **13.27 U/L lower** (21.39 lower to 5.16 lower)	⨁◯◯◯ Very low	IMPORTANT
**Aspartate transferase**												
5	randomized trials	serious^a^	very serious^d^	not serious	serious^d^	none	898	560	-	MD **7.02 U/L lower** (11.26 lower to 2.78 lower)	⨁◯◯◯ Very low	IMPORTANT
**Homeostatic model of assessment for insulin resistance**												
3	randomized trials	serious^a^	serious^e^	not serious	not serious	none	745	587	-	MD **2.07 lower** (2.69 lower to 1.46 lower)	⨁⨁◯◯ Low	IMPORTANT

**CI**: confidence interval; **MD**: mean difference,

Explanations:

^a^. Unclear or high risk of bias,

^b^. Substantial heterogeneity with a I^2^ = 65%,

^c^. Substantial heterogeneity with a I^2^ = 95%,

^d^. Substantial heterogeneity with an I^2^ = 88%,

^e^. Substantial heterogeneity with a I^2^ = 69%.

**Fig 4:**
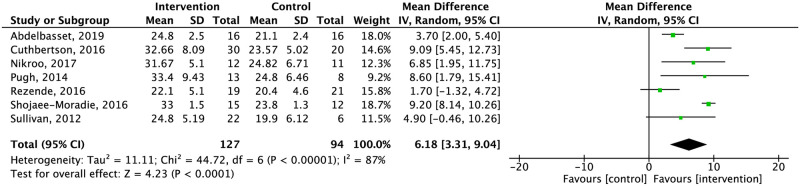
Effect of exercise alone compared to the control in oxygen consumption.

*Oxygen consumption*. Six studies assessed oxygen consumption in NAFLD patients [39, 40, 45, 46, 48, 49] and one study in NASH [44] assessed patients after exercise alone. Among these, six were supervised. None of the studies assessing diet or the combination of diet and exercise evaluated this outcome (Table 2). Exercise alone significantly increased oxygen consumption compared to the control group (low quality of evidence) (Fig 4).

*HOMA-IR*. Sixteen studies assessed the effects of exercise, diet, or the combination of both in HOMA-IR. Exercise alone and the combination of exercise and diet significantly reduced HOMA- IR compared to the control group (low to moderate quality of evidence). When we compared with different control groups, exercise reduced HOMA-IR only against standard care (-0.48 CI 95% -0.92, -0.02 I^2^ = 28%) (S3–S5 Figs).

*Intrahepatic lipids*. Fourteen studies measured the effect of exercise alone on intrahepatic lipids, assessed by H-MRS [40–42, 45, 48, 48, 53], MRI [38, 39, 43], US [44, 47, 50, 52] and Fibroscan CAP (46). Four studies researched the effect of diet alone measured by US [56, 58, 59] and MRI [57]. Six of eight studies assessing the effect of combined interventions evaluated IHL with H-MRS [24, 64], NAS score [63, 65], US [62] and liver-spleen ratio by computed tomography [66].

**Fig 5:**
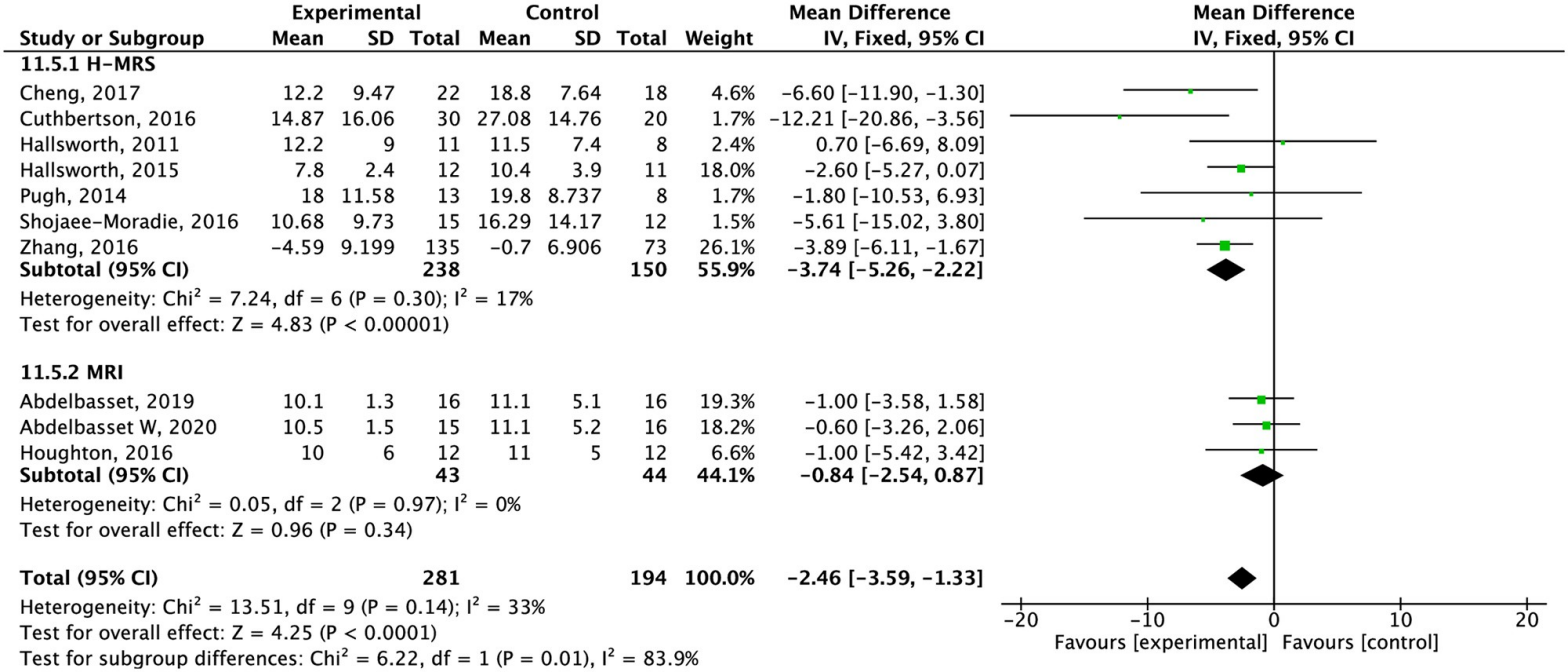
Effect of exercise alone compared to the control group in intrahepatic lipids assessed by H-MRS and MRI.

Exercise significantly reduced intrahepatic lipids compared to the control group assessed by H-MRS but there were no differences in MRI assessment (moderate quality of evidence) (Fig 5). With US assessment, exercise showed to improve IHL from baseline to the end of the intervention in three of the four studies. Zelber-Sagi et al. [52] showed that resistance training produced a reduction of 0.25 ± 0.37 on the hepatorenal index score. Takahashi et al. [50] showed reduction in steatosis liver grade too, after resistance training protocol (2.00 ± 0.82 vs. 1.55 ± 0.71, p = 0.001). Shamsoddini et al. [47] showed that aerobic exercise and resistance exercise also significantly reduced hepatic fat grade too.

**Fig 6:**
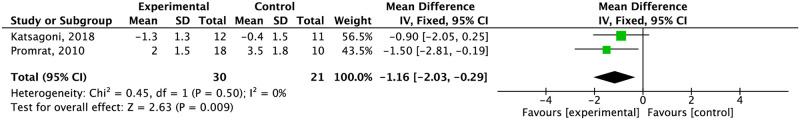
Effect of exercise plus diet compared to the control group in intrahepatic lipids assessed by NAS score.

On the NAS score, the combination of diet and exercise showed a significant difference compared to the control group (Fig 6). Cheng et al. [61] evaluated the effects of diet plus exercise on intrahepatic lipids with H-MRS and it showed an important reduction compared to the group with no treatment (-47,9% vs 20,9% increment on the control group by the end of intervention).

In diet alone interventions, Nourian et al. [58] assessed the effect of enhancing the intake of fruits, vegetables, complex carbohydrates, white meat, and fish while avoiding unhealthy fats and refined carbohydrates. They compared that with usual care and showed that the severity of liver steatosis, evaluated with ultrasonography, improved significantly in the intervention group compared to the control group (p = 0.025). Marin-Alejandre et al. [57] assessed the effect of the FliO diet vs the AHA diet and showed that both diets improved liver steatosis compared to their baseline values (p <0.0001 on both) without significant differences between them (p = 0.706). These differences were assessed with MRI.

*Alanine transaminase*. Twenty studies evaluated the effect of exercise in ALT levels, five the effect of diet alone, and five studies the effect of diet combined with exercise.

**Fig 7:**
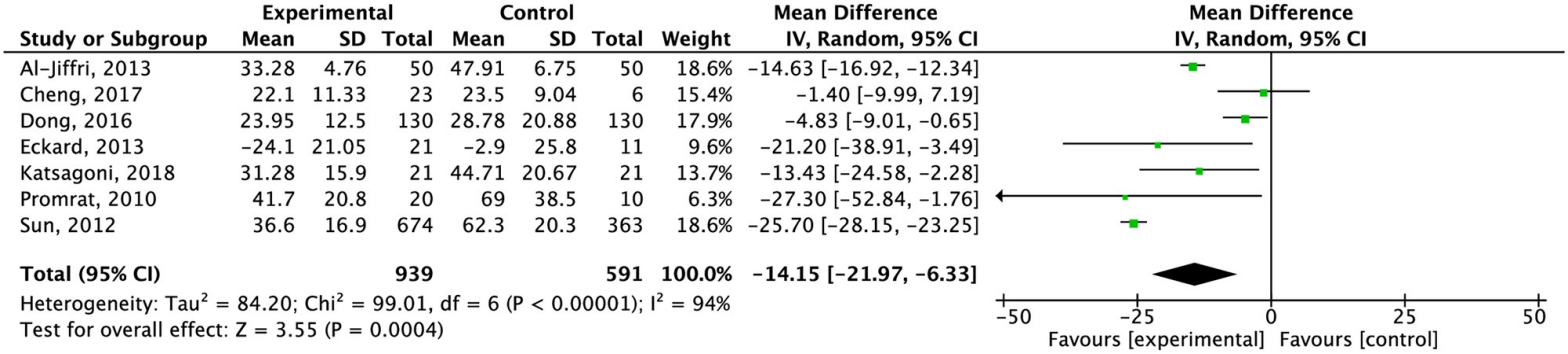
Effect of exercise plus diet compared to the control group in ALT concentration.

Combination of exercise and diet significantly reduced ALT levels compared to the control group (Fig 7) (moderate and very low quality of evidence respectively). Compared specifically to education and standard care, the effect of combined intervention was higher against education. The heterogeneity remained higher after separated by type of control group (I^2^ = 97%) (S6 Fig).

**Fig 8:**
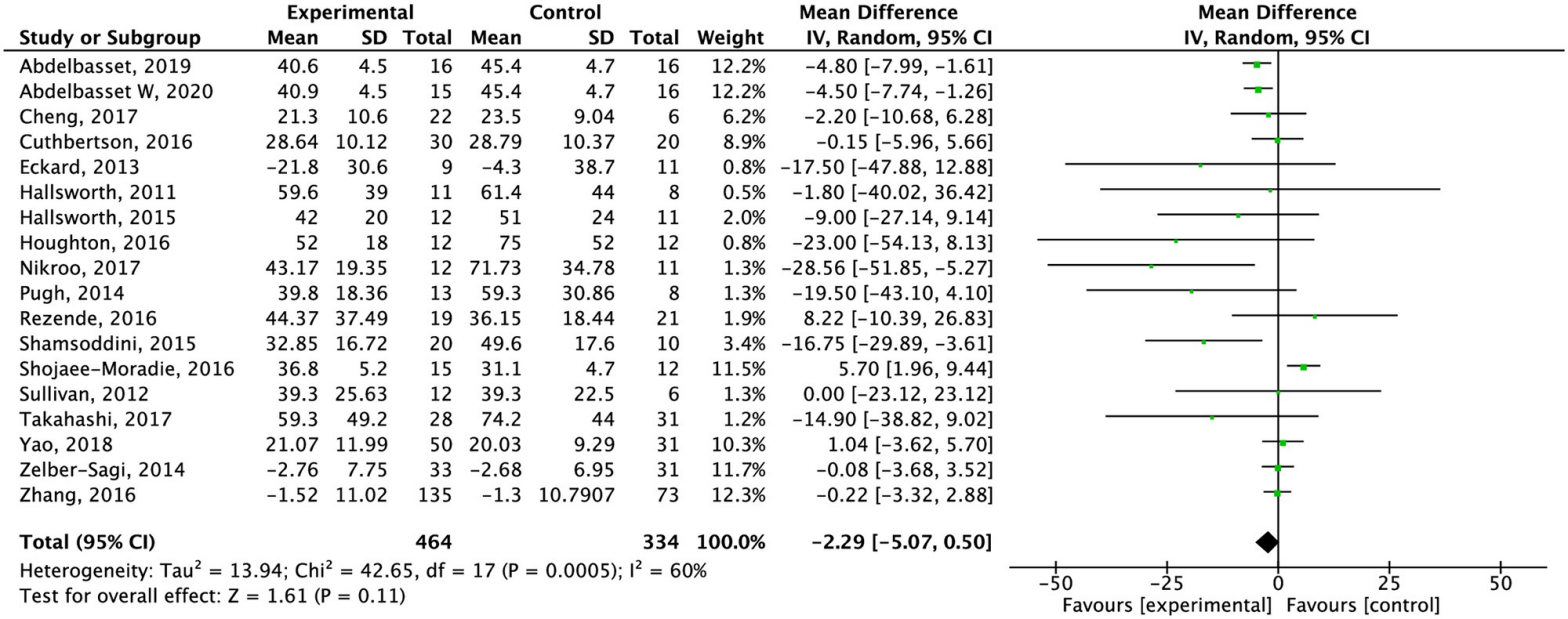
Effect of exercise alone compared to the control group in ALT concentration.

Exercise alone did not significantly reduce ALT levels (low quality of evidence) (Fig 8). Compared with different types of controls groups, the effect of exercise in ALT levels was significant against normal physical activity and standard care (-17.55 CI% 30.69, -4.41 and -4.94 CI 95% -7.17, -2.7 I^2^ = 0% respectively) (S7 Fig).

Individually, all studies assessing diet alone reduced ALT levels within their intervention groups, and three of them showed a significant difference with their control group. The DASH diet reduced ALT to 8.4 ± 16.5 U/L compared to the control group where ALT increased on 3.8 ± 23.9 U/L [59]. The FliO diet and the AHA diets significantly improve ALT in almost 10 U/L without a significant difference between groups (p = 0.474) [57]. Alternate-day calorie restriction significantly reduces ALT compared to the usual diet (59.17 vs. 84.33 IU/L, P = 0.001). Ghetti et al. [56] showed that individualized diet compared to nutritional orientation did not reduce ALT levels significantly.

*Aspartate transaminase*. Twenty-one studies assessed the effect of lifestyle interventions in AST levels. Fourteen considered exercise alone, five assessed the effect of diet alone and five evaluated the effects of diet plus exercise.

**Fig 9:**
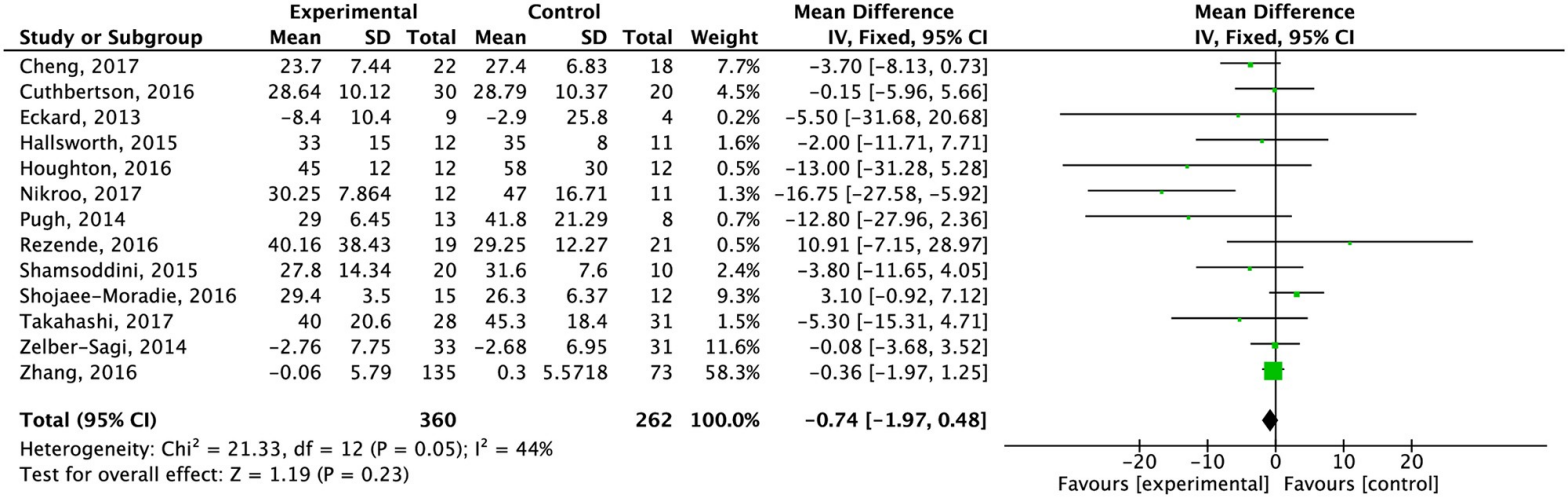
Effect of exercise compared to the control group in AST concentration.

**Fig 10:**
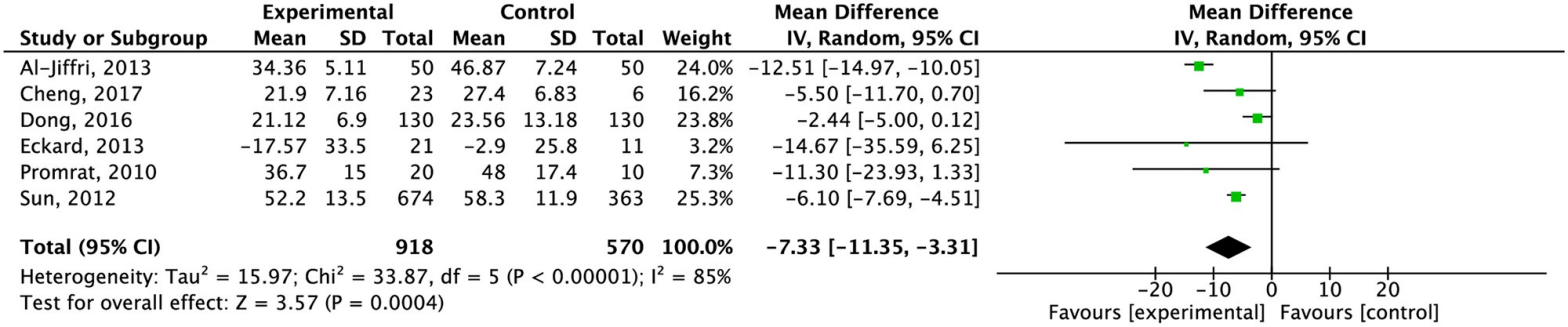
Effect of exercise plus diet compared to the control group in AST concentration.

Exercise alone reduced AST levels. However, this reduction was not significant compared to the control group (Fig 9) (moderate quality of evidence). After separating by type of control, the effect remained no significant (S8 Fig). AST levels decreased significantly compared to the control group when exercise was combined with diet (-7.33 CI 95% -11.35, -3.31) (Fig 10). When it was compared specifically with standard care and education about a healthy lifestyle, the effect of a diet and exercise combination was higher against standard care on AST levels (-10.76 CI 95% -10.76, -8.74 I^2^ = 75%) (S9 Fig).

Diet studies showed significant differences within the intervention group (Table 3). Only in the study of Nourian et al. [58], was AST significantly lower after the intervention against the control group (23.90 ± 1.45 vs 30.80 ± 3.20 respectively, p<0.001).

*Analysis by length of intervention*. Exercise alone significantly reduced ALT levels (-5.05 CI 95% -7.48, -2.62) and HOMA-IR (-0.96 CI 95% -1.60, -0.32) when compared to conventional treatment in periods of time equal to or shorter than eight weeks. After two months, weight and IHL levels decreased significantly compared to the conventional treatment and after 24 weeks only changes in IHL remained significant. AST reduction was not significant after analyzing the duration of the intervention (S10–S14 Figs).

The combination of diet and exercise significantly reduced ALT (-14.63 CI 95% -16.92, -12.34) and AST (-12.51 CI 95% -14.97, -10.05) within eight and 24 weeks. AST changes remained significant after 24 weeks (-4.84 CI 95% -7.33, -2.36). Independent of duration of intervention, weight did not significantly change (S15–S17 Figs).

*Analysis by supervision or no supervision*. Supervised exercise compared to non-supervised exercise considerably reduced weight (-1.56 CI 95% -2.24, -0.88) and IHL (-3.68 CI 95% -6.97, -0.39) considerably in patients with NAFLD. In contrast, ALT (-5.27 CI 95% -7.44, -3.1) and HOMA-IR (-0.92 CI 95% -1.48, -0.37) decreased in studies in which exercise was non-supervised. AST was not significant in supervised or non-supervised exercise (S18–S22 Figs).

## Discussion

In this meta-analysis we assessed the effects of lifestyle changes in patients with NAFLD. We analyzed the effects of exercise alone, diet alone or the combination of both and performed a subgroup analysis whenever there were enough studies available to assess the effect of the duration of the intervention and whether it was supervised. Considering that control groups were heterogeneous, we additionally analyzed data by grouping control groups as standard care, education, or advice about NAFLD and healthy lifestyle and maintaining daily dietary or physical activity habits.

Compared to the control group, exercise alone did not reduce weight. However, after grouped by type of control, exercise reduced weight compared to maintaining physical daily activities and education about a healthy lifestyle (MD: -8.06 kg and MD: -1.85 kg respectively), HOMA-IR (MD: -0.46), oxygen consumption (MD: 6.18 mlO_2_/kg/min) and intrahepatic lipids (-3.74% assessed by H-MRS). The combination of diet and exercise reduced weight only compared to education about healthy lifestyle (-2.82 kg), HOMA-IR compared to the control group (MD: -2.07), IHL assessed by NAS score (MD: -1.16) and liver enzymes (AST: MD: -7.33 U/L; ALT: MD: -14.15 U/L).

Exercise alone improved HOMA-IR and ALT levels within eight weeks, whereas diet alone reduced transaminases. On interventions between 8 to 24 weeks, exercise reduced weight and IHL, and the combination of diet and exercise reduced both transaminases. After 24 weeks, exercise only significantly improved IHL and the combination of exercise and diet reduced only AST.

Studies that only assessed exercise were supervised, and therefore we grouped them to compare supervision and no supervision. Supervised exercise significantly reduced weight and IHL. In contrast, no supervised exercise reduced ALT levels and HOMA-IR.

The overall level of evidence in this review ranged from very low to moderate. On one side, being unclear, or having a high risk of bias was judged for most trials in allocation concealment, blinding of participants and blinding of outcome assessment. On the other side we considered serious inconsistency in most outcomes due to the high heterogeneity between trials.

NAFLD, metabolic syndrome, T2DM and dyslipidemia are pathologies associated with lifestyle decisions, being a subgroup of well-known non-communicable diseases (NCDs). These are developed by complex interactions between environmental, physiological, genetic, and behavioral factors [67] and can jeopardize a patient’s health, affecting their daily activities, limiting their functionality, and consequently leading to a poorer quality of life [68], predisposing them to a greater risk of developing cardiovascular diseases (CVDs). In fact, these are one of the four major NCDs and the leading cause of global burden diseases associated with premature deaths. NAFLD and the health conditions mentioned share risk factors such as unhealthy diet, low physical activity and especially obesity. Therefore, their main therapy must include lifestyle changes [68].

Weight loss is an important goal to achieve in these conditions due to its relationship with obesity [69]. Mild weight loss improves blood pressure and metabolic parameters whereas moderate loss enhances these benefits, reducing the risk of CVDs [70]. In NAFLD, modest weight loss (>5%) improved liver steatosis, enzyme concentrations and other extrahepatic conditions [71]. When losses are greater than 10% there is a considerable reduction of steatosis and a significant regression on NASH and fibrosis [5]. Additionally, Tapper et al. [72] reported that weight loss is associated with better QoL in patients with NAFLD, as individuals who lost weight improved the overall CLDQ domains compared to non-improvers.

In our study we assessed QoL as the primary outcome as it reinforces the concept that illness can impact self-perception and life satisfaction overall [68]. However, only two studies assessed quality of life after lifestyle changes by CLDQ and SF-36, improving all domains such as physical function, vitality, and role limitation due to physical health respectively [39, 44]. Only one of these showed that reduction of body mass index was correlated with improvements in quality of life, as patients exposed to high intensity interval training improved both in contrast to control groups who did not change body mass index nor QoL [39]. Similar results were observed in Hickman et al., in which patients with chronic liver diseases reported better QoL after permanent lifestyle modifications, especially those who lost weight in the first three month intervention and maintained this loss after 15 months of follow up [73].

Accordingly, combined exercise and diet would have the greatest effect on weight. Our results showed that it only reduced 1.54 kg on average, which was not significant when compared to the overall control group. However, when compared to different control groups, the reduction was greater and significant against only educate patients about a healthy lifestyle (-2.82 kg). Exercise alone also reduced weight compared to education, however this effect was smaller (-1.85 kg). We could not estimate the pool effect of diet alone in weight loss, because studies were highly heterogeneous, however, they individually all showed significant reduction of body weight. Our results are in line with other studies as the combination of both interventions is better than diet or exercise alone. In overweight and obese postmenopausal women, combined interventions produced a larger weight loss (10.8%) than diet (8.5%) or exercise (0.8%) [74] whereas, in older overweight and obese adults, diet combined with exercise reduced weight by 11.3%, diet alone by 9.5% and exercise alone by 2% [75]. Diet plays an important role because it reduces energy intake, leading to negative energy balance and weight loss, and thus reduced energy expenditure. This reduction can be compensated by adding exercise as it increases metabolic rate and energy expenditure [76]. Together diet and exercise can enhance weight loss and maintain it in the long-term [76]; this is the most recommended strategy to achieve weight loss in different metabolic diseases guidelines [11, 77]. Contrary to this, there are some studies sustaining that exercise may not enhance weight loss but gives other important benefits. Larson-Meyer et al. [78] showed that after six months of calorie restriction and calorie restriction combined with aerobic exercise, both groups reduced weight by 10% as well as fat mass by 25% in patients with NAFLD, but adding exercise increased oxygen consumption, insulin sensitivity, diastolic pressure, and total cholesterol. The same results were seen in the study by Redman et al. [79] in which a calorie restriction of 25% compared to a calorie restriction of 12,5% with an energy expenditure of 12.5% achieved with exercise, produced similar body composition changes. However, in the combined group, participants also improved oxygen consumption and insulin sensitivity.

Based on this, exercise may not have an important effect on weight loss; but, it has several other metabolic benefits [70]. Cardiorespiratory fitness (CRF), which is a physiological indicator of maximum oxygen consumption in response to metabolic demands, is an independent predictor of cardiovascular disease, all-cause mortality [80] and disease-specific mortality [81, 82]. Croci et al. [83] also demonstrated that low CRF in NAFLD patients is a predictor of premature death and all-cause mortality as well. Besides, this can also predict liver fat modifications and the resolution of the disease [84]. It has been demonstrated that exercise increases CRF [85, 86]. A meta-analysis concluded that moderate to vigorous aerobic exercise improved CRF by 11.8% in the exercise group compared to the control group [87]. Our systematic review supported the positive effect of exercise in CRF, as exercise increased oxygen consumption in 6.18 ml O2•kg^−1^•min^−1^ compared to the control group in patients with NAFLD. These results are relevant as one metabolic equivalent of task (MET) improvement which corresponds to 3.5 mlO2•kg^−1^•min^−1^, reduce all-cause mortality risk, sudden cardiac death risk [88] triglycerides, fasting plasma glucose [80], insulin, HOMA-IR, and HDL-c [89]. Therefore, our results indicate that exercise improves 1.79 METs, improving CRF and consequently several other metabolic parameters. Nevertheless, this result should be validated through prospective long-term studies.

Insulin resistance plays an important role in the development and progression of NAFLD [1] and constitutes one of the major determinants of CVDs [81]. We assessed the effect of lifestyle interventions in HOMA-IR. The combination of diet and exercise decreased HOMA-IR by 2.07 points, and exercise alone reached a difference of 0.46 compared to the control group. Similarly, Katsagoni et al. [7] concluded that the combination of exercise and diet produce large effects on HOMA-IR (SMD: -1.17) compared to exercise alone (SMD: -0.76). In another systematic review of lifestyle changes and NAFLD the results varied since in some studies the combination of both interventions did not reduce HOMA-IR, whereas in others there was a major reduction of up to 40% [13].

In terms of liver parameters, the combination of both interventions decreased ALT and AST levels (ALT MD: -13.27; AST MD: -7.02). Studies showed improvements in both enzymes in diet intervention, too. However, the effect on liver enzymes, importantly differs among studies as some of them only showed differences within the intervention group. A novel systematic review and meta-analysis by Houttu et al. [90] showed that hypocaloric diets reduce ALT levels SMD: −1.09, 95% CI: −1.49, −0.69) as well as AST levels compared to the control group (SMD: −0.75, 95% CI: −1.27, 0.23). Exercise by itself did not reduce liver enzymes, differing from the results showed by Orci et al. [91] (ALT: weighted mean difference [WMD] –3.30 IU/L; 95% CI, –5.57 to –1.04; AST: WMD –4.85 IU/L; 95% CI, –8.68 to –1.02).

Due to the heterogeneity of assessment on IHL, we decided to assess the effect of exercise and combined interventions by type of measurement method. Assessed with H-MRS, exercise alone reduced IHL significantly by 3,74%. With MRI, exercise did not change IHL significantly. We could only pool two studies considering diet plus exercise as an intervention, which assessed IHL with NAS score. Compared to the control group, they reduced steatosis by 1.16%. We did not pool the rest of the studies as they used US, fibroscan CAP, and other methods to assess IHL. Our results highlight the variety of techniques used to assess liver fat, each with different advantages, disadvantages, and cut-off points. According to Castera et al. [92], despite the significant progress made in the noninvasive assessment of liver fat, there are still needs to cover like validation of quality criteria, and cost-effectiveness of sequential use of these techniques, among others. We also must consider that, even though our results showed significant differences, in practice, these changes may not be detectable by MRI or H-MRS, so we could not draw a solid conclusion about the specific change in IHL.

We realized that other reviews did not assess the effect of the duration of the intervention on NAFLD. Therefore, we decided to include it in our review. On the one hand, exercise may reach better results when performed for longer periods of time (> 8 weeks and 24 weeks), as it can reduce liver fat and weight compared to the conventional treatment. On the other hand, diet improved aminotransferases in a short period of time (< 8 weeks). However, this effect is not sustained over time. According to Hallsworth et al. [93], most studies assessing lifestyle changes are relatively short, ranging from 8 to 12 weeks in which some positive effects on NAFLD can be evaluated within this time, which could explain our results. Individually, all studies concluded that these interventions improved liver fat and other metabolic parameters [53, 24, 62]. Nevertheless, we cannot state that interventions longer than 24 weeks have beneficial effects on NAFLD compared to the control group as only three of the included studies exceed that time. Wong et al. [35] stated that lifestyle changes improved body weight and IHL and reduced or normalized ALT in in obese and non-obese patients after a year of intervention. After A six-year follow-up, both groups had regained weight, but less than the control group. Besides, non-obese participants maintained their ALT levels while obese people rebounded to their baseline levels. These changes can be explained by low adherence or unsustainable changes through time [59]. Supervision is a relevant factor that can improve patient adherence and motivation. According to Hunter et al. [94] supervised strategies (specifically supervised exercise) improved health, fitness, and QoL in several health conditions. In our study we evaluated that supervised exercise improved weight loss and IHL in NAFLD patients. Similarly, Nikolai et al. [95] concluded that supervision leads to a larger decrease in body weight and body fat (8 kg and 6.2 kg respectively) compared to a non-supervised control group (2.8 kgs. and 1.7 kg respectively). The large Italian Diabetes and Exercise Study (IDES) [96] states that supervised exercise twice a week exceeds the physical fitness and CVD risk profile in patients with T2DM. Supervision may enhance exercise objectives as it can encourage progression and changes to the routines, increase the intensity of the program, and promote adherence and exercise self-efficacy. However, these results have been studied in the short term. Even though they are effective, there is no evidence to support its benefits in the long term.

This meta-analysis has limitations. First, there is a broad heterogeneity between included studies in terms of NAFLD diagnostic methods, type of participants and intervention protocols. Because it is unethical to not inform and give standard recommendations regarding diet and exercise to the control group, several types of control groups were included such as education or advice about NAFLD and a healthy lifestyle, daily normal physical activities, standard care even calorie restriction, flexibility training and specific dietary patterns such as the AHA diet, which could alter our results. We grouped by control group and assessed the effect of exercise and the combination of diet and exercise against a specific control, showing some significant results with less heterogeneity between studies. However, this supports the fact that lifestyle changes strategies are heterogeneous, and there is still no consensus about the optimal treatment. Moreover, these differences did not allow us to assess or concluded about the effect of diet alone, as we only included six studies and all of them were different. Second, there was unavailable data in twelve studies. We tried to reach the authors, but ten of them did not reply. Therefore, data was calculated based on formulas, which could also alter our results. Finally, we included an important number of trials assessed with the Cochrane Risk of Bias Tool, which had unclear or high risk of bias in some domains and leading to a decreased level of evidence.

Despite these limitations, we highlight that our results are concordant with previous studies. Therefore, lifestyle modifications in NAFLD in terms of diet and exercise interventions improve metabolic parameters and liver function tests. Besides, this review included QoL and oxygen consumption within its outcomes, which were not included in other lifestyle intervention meta-analyses. We can conclude that lifestyle modifications can improve QoL and increase oxygen consumption, decreasing the risk of cardiovascular diseases and mortality in these patients. Moreover, we studied the effects of these interventions in terms of their length, assessing them in the short and long term. Additionally, we determined that supervised exercise is more beneficial in weight management and IHL in NAFLD patients, which is consistent with the conclusions of other studies that assessed weight loss, physical activity, and fitness [97, 98].

To continue expanding our knowledge regarding this topic, there should be more prospective studies researching the effects of lifestyle modifications in the long term, as there are few regarding this topic in the current literature. Another interesting approach is to assess the effect of supervised exercise in the long term on these patients.

## Conclusion

Lifestyle modifications are effective in the treatment of NAFLD. Diet, exercise, or the combination of both are beneficial in the treatment of NAFLD. Dietary and exercise changes combined are superior to these interventions alone improving metabolic parameters and liver function tests. Exercise may add other benefits in patients with NAFLD as it increases cardiorespiratory function, which may be a protector of cardiovascular diseases. Better results may be achieved after longer periods of time (> 8 weeks) and with supervised interventions. However, these ideas currently lack enough evidence to support them.

## Supporting information

S1 File PRISMA checklist. (DOCX)

S2 File Search strategy for PubMed, embase and CENTRAL. (DOCX)

S1 Table Risk of bias assessment. (DOCX)

S1 Fig Effect of exercise on weight loss by type of the control group. (TIF)

S2 Fig Effect of exercise plus diet on weight loss by type of the control group. (TIF)

S3 Fig Effect of exercise alone compared to the control group in HOMA-IR. (TIF)

S4 Fig Effect of exercise plus diet compared to the control group in HOMA-IR. (TIF)

S5 Fig Effect of exercise alone on HOMA-IR by type of control group. (TIF)

S6 Fig Effect of exercise plus diet on ALT levels by type of control group. (TIF)

S7 Fig Effect of exercise alone on ALT levels by type of control group. (TIF)

S8 Fig Effect of exercise alone on AST levels by type of control group. (TIF)

S9 Fig Effect of exercise plus diet on AST levels by type of control. (TIF)

S10 Fig Effect of exercise on ALT levels by length of intervention. (TIF)

S11 Fig Effect of exercise on HOMA-IR by length of intervention. (TIF)

S12 Fig Effect of exercise alone on weight loss by length of intervention. (TIF)

S13 Fig Effect of exercise alone on IHL by length of intervention. (TIF)

S14 Fig Effect of exercise alone on AST levels by length of intervention. (TIF)

S15 Fig Effect of exercise plus diet on ALT levels by length of intervention. (TIF)

S16 Fig Effect of exercise plus diet on AST levels by length of intervention. (TIF)

S17 Fig Effect of exercise plus diet on weight loss levels by length of intervention. (TIF)

S18 Fig Effect of exercise alone on weight loss by supervision. (TIF)

S19 Fig Effect of exercise alone on IHL by supervision. (TIF)

S20 Fig Effect of exercise alone on ALT levels by supervision. (TIF)

S21 Fig Effect of exercise alone on HOMA-IR by supervision. (TIF)

S22 Fig Effect of exercise alone on AST by supervision. (TIF)
