# Supplementary material for: Lifestyle changes in patients with non-alcoholic fatty liver disease: A systematic review and meta-analysis
Source: PLoS One. 2022 Feb 17;17(2):e0263931. doi: 10.1371/journal.pone.0263931 (PMC8853532; doi:10.1371/journal.pone.0263931)
Supplement: S2 File — (DOCX) [file pone.0263931.s002.docx]

**Search strategy**

Search date: May 18^th^, 2020

***Pubmed*:**

Hit: 3361

(NAFLD* OR NASH*[tiab] OR "non-alcoholic" OR "non alcoholic" OR nonalcoholic* OR "fatty liver" OR "fatty-liver" OR "liver steatosis" OR "liver-steatosis" OR steatohepatit* OR "hepatic steatosis" OR "hepatic-steatosis") AND (exercise* OR (physical* AND activit*) OR training* OR strength* OR aerobic* OR endurance* OR sport OR fitness* OR yoga* OR yogi* OR pranayam* OR asana* OR dhyana* OR hatha* OR iyengar* OR flexibility* OR hydrotherapy* OR Taiji* OR "Tai ji" OR "Tai-ji" OR Taichi* OR "Tai Chi" OR "Tai-Chi" OR "Qi gong" OR "Qi-gong" OR Qigong* OR pilates* OR diet OR diets* OR dietetic* OR dietary* OR ((calori* OR energy* OR fat) AND restrict*) OR nutrition* OR "low-calorie" OR "low-energy" OR hypocaloric OR "energy-restricted" OR "calorie-restricted" OR "low-fat" OR "reduced-fat" OR "moderate-fat" OR "fat-restricted" OR "lower-fat" OR "carbohydrate-restricted" OR "low-carbohydrate" OR "low-carb" OR lifestyle*) AND ((randomized controlled trial [pt] OR controlled clinical trial [pt] OR randomized [tiab] OR placebo [tiab] OR drug therapy [sh] OR randomly [tiab] OR trial [tiab] OR groups [tiab]) NOT (animals [mh] NOT humans [mh]))

***Embase* (Elsevier)**

Hits: 1500

Filters: Meta-analysis, controlled clinical trial, randomized controlled trial

(NAFLD* OR NASH*:ti,ab OR "non-alcoholic" OR "non alcoholic" OR nonalcoholic* OR "fatty liver" OR "fatty-liver" OR "liver steatosis" OR "liver-steatosis" OR steatohepatit* OR "hepatic steatosis" OR "hepatic-steatosis") AND (exercise* OR (physical* AND activit*) OR training* OR strength* OR aerobic* OR endurance* OR sport OR fitness* OR yoga* OR yogi* OR pranayam* OR asana* OR dhyana* OR hatha* OR iyengar* OR flexibility* OR hydrotherapy* OR Taiji* OR "Tai ji" OR "Tai-ji" OR Taichi* OR "Tai Chi" OR "Tai-Chi" OR "Qi gong" OR "Qi-gong" OR Qigong* OR pilates* OR diet OR diets* OR dietetic* OR dietary* OR ((calori* OR energy* OR fat) AND restrict*) OR nutrition* OR "low-calorie" OR "low-energy" OR hypocaloric OR "energy-restricted" OR "calorie-restricted" OR "low-fat" OR "reduced-fat" OR "moderate-fat" OR "fat-restricted" OR "lower-fat" OR "carbohydrate-restricted" OR "low-carbohydrate" OR "low-carb" OR lifestyle*)

***CENTRAL***

Hits: 1751

Filters: Trials

(NAFLD* OR (NASH*):ti,ab OR "non-alcoholic" OR "non alcoholic" OR nonalcoholic* OR "fatty liver" OR "fatty-liver" OR "liver steatosis" OR "liver-steatosis" OR steatohepatit* OR "hepatic steatosis" OR "hepatic-steatosis") AND (exercise* OR (physical* AND activit*) OR training* OR strength* OR aerobic* OR endurance* OR sport OR fitness* OR yoga* OR yogi* OR pranayam* OR asana* OR dhyana* OR hatha* OR iyengar* OR flexibility* OR hydrotherapy* OR Taiji* OR "Tai ji" OR "Tai-ji" OR Taichi* OR "Tai Chi" OR "Tai-Chi" OR "Qi gong" OR "Qi-gong" OR Qigong* OR pilates* OR diet OR diets* OR dietetic* OR dietary* OR ((calori* OR energy* OR fat) AND restrict*) OR nutrition* OR "low-calorie" OR "low-energy" OR hypocaloric OR "energy-restricted" OR "calorie-restricted" OR "low-fat" OR "reduced-fat" OR "moderate-fat" OR "fat-restricted" OR "lower-fat" OR "carbohydrate-restricted" OR "low-carbohydrate" OR "low-carb" OR lifestyle*)
