# Supplementary material for: Lifestyle changes in patients with non-alcoholic fatty liver disease: A systematic review and meta-analysis
Source: PLoS One. 2022 Feb 17;17(2):e0263931. doi: 10.1371/journal.pone.0263931 (PMC8853532; doi:10.1371/journal.pone.0263931)
Supplement: S1 Table — (DOCX) [file pone.0263931.s003.docx]

| **Author, year** | **Random sequence generation (selection bias)** | **Allocation concealment (selection bias)** | **Blinding of participants and personnel (performance bias)** | **Blinding of outcome assessment (detection bias)** | **Incomplete outcome data (attrition bias)** | **Selective reporting (reporting bias)** |
| --- | --- | --- | --- | --- | --- | --- |
| Abdelbasset, 2019 | Low Risk | Low Risk | Unclear Risk | Low Risk | Low Risk | Low Risk |
| Abdelbasset, 2020 | Low Risk | Low Risk | Low Risk | Low Risk | Low Risk | Low Risk |
| Al-Jiffri, 2013 | Low Risk | Unclear Risk | Unclear Risk | Unclear Risk | Low Risk | Unclear Risk |
| Arab, 2017 | Low Risk | Unclear Risk | Low Risk | Low Risk | Low Risk | High Risk |
| Cai, 2019 | Low Risk | Low Risk | Unclear Risk | Unclear Risk | Low Risk | Low Risk |
| Cheng, 2017 | Low Risk | Low Risk | Low Risk | Low Risk | Low Risk | Low Risk |
| Cuthbertson, 2015 | Low Risk | High Risk | High Risk | Unclear Risk | High Risk | Low Risk |
| Dong, 2016 | Low Risk | Unclear Risk | Unclear Risk | Unclear Risk | Low Risk | Low Risk |
| Eckard, 2013 | Low Risk | Low Risk | Unclear Risk | Low Risk | Low Risk | Low Risk |
| Ghetti, 2019 | Low Risk | Low Risk | High Risk | High Risk | Low Risk | Low Risk |
| Hallsworth, 2011 | High Risk | Unclear Risk | Unclear Risk | Unclear Risk | Low Risk | Low Risk |
| Hallsworth, 2015 | Low Risk | Unclear Risk | High Risk | High Risk | Low Risk | Low Risk |
| Houghton, 2016 | Low Risk | Unclear Risk | Unclear Risk | Unclear Risk | Low Risk | Low Risk |
| Johari, 2019 | Low Risk | Unclear Risk | Unclear Risk | Unclear Risk | High Risk | Low Risk |
| Katsagoni, 2018 | Low Risk | Unclear Risk | High Risk | Low Risk | Low Risk | Low Risk |
| Marin-Alejandre, 2019 | Unclear Risk | Unclear Risk | Low Risk | Low Risk | Low Risk | Low Risk |
| Nikroo, 2017 | High Risk | Unclear Risk | Unclear Risk | Unclear Risk | Low Risk | Low Risk |
| Nourian, 2020 | Low Risk | Low Risk | High Risk | Unclear Risk | Low Risk | Low Risk |
| Pugh, 2014 | Low Risk | Unclear Risk | High Risk | High Risk | Low Risk | Low Risk |
| Razavi, 2015 | Low Risk | Low Risk | Low Risk | Low Risk | Low Risk | Low Risk |
| Rezende, 2016 | Low Risk | Low Risk | Unclear Risk | Unclear Risk | Low Risk | Low Risk |
| Shamsoddini, 2015 | Unclear Risk | Unclear Risk | Unclear Risk | Unclear Risk | Low Risk | Low Risk |
| Shojaee-Moradie, 2016 | Low Risk | Unclear Risk | High Risk | High Risk | Low Risk | Low Risk |
| Sullivan, 2012 | Low Risk | Low Risk | High Risk | High Risk | Low Risk | Low Risk |
| Sun, 2012 | Low Risk | Low Risk | Low Risk | Low Risk | Low Risk | Low Risk |
| Takahashi, 2015 | High Risk | Unclear Risk | Unclear Risk | Unclear Risk | Low Risk | Low Risk |
| Yao, 2018 | Low Risk | Unclear Risk | Unclear Risk | Low Risk | Low Risk | Low Risk |
| Zelber-Sagi, 2014 | Low Risk | Low Risk | High Risk | Low Risk | Low Risk | Low Risk |
| Zhang, 2016 | Low Risk | Unclear Risk | High Risk | Low Risk | Low Risk | Low Risk |
