# Supplementary figures and images for: Lifestyle changes in patients with non-alcoholic fatty liver disease: A systematic review and meta-analysis
Source: PLoS One. 2022 Feb 17;17(2):e0263931. doi: 10.1371/journal.pone.0263931 (PMC8853532; doi:10.1371/journal.pone.0263931)

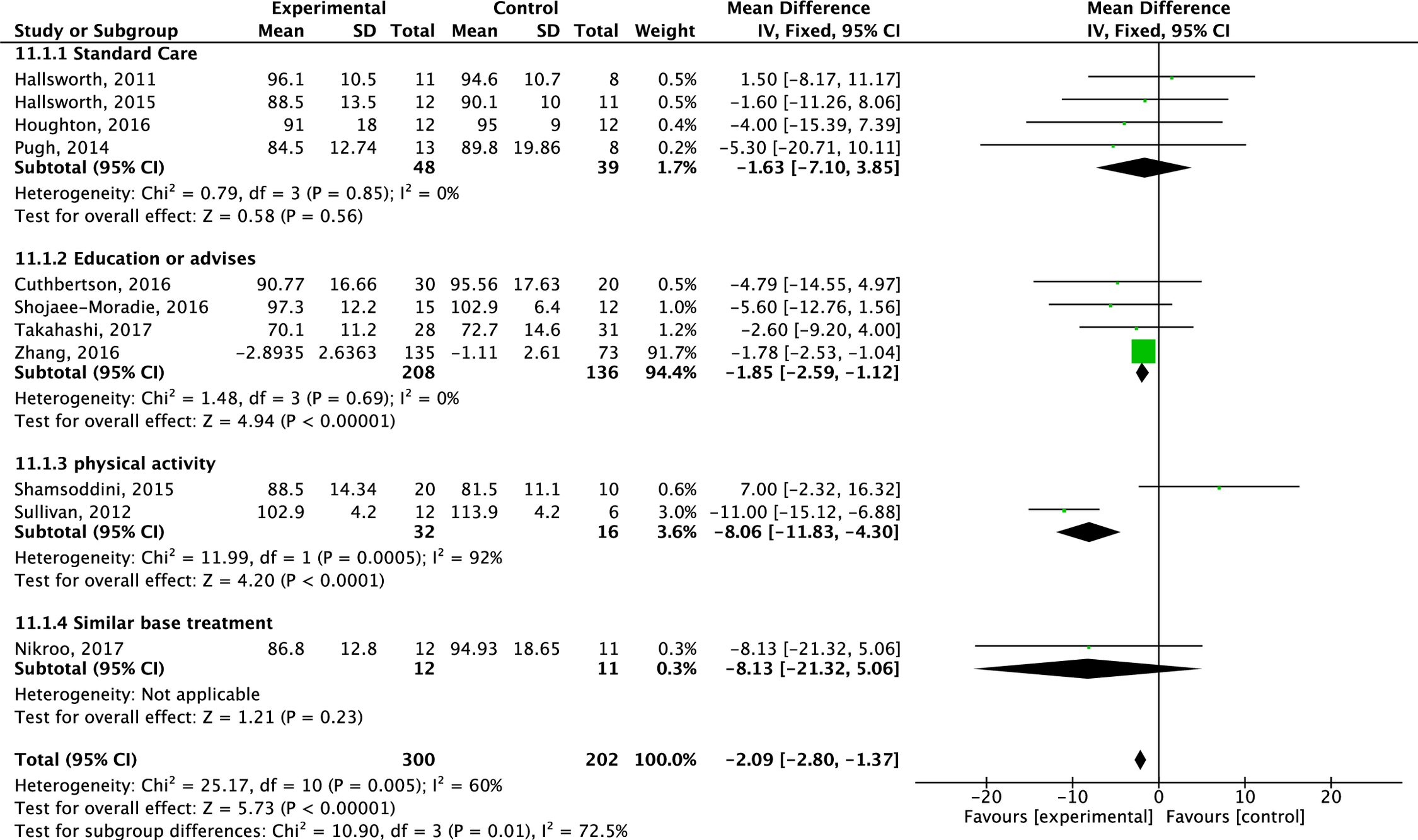

Supplement: S1 Fig — (TIF) [file pone.0263931.s004.tif]

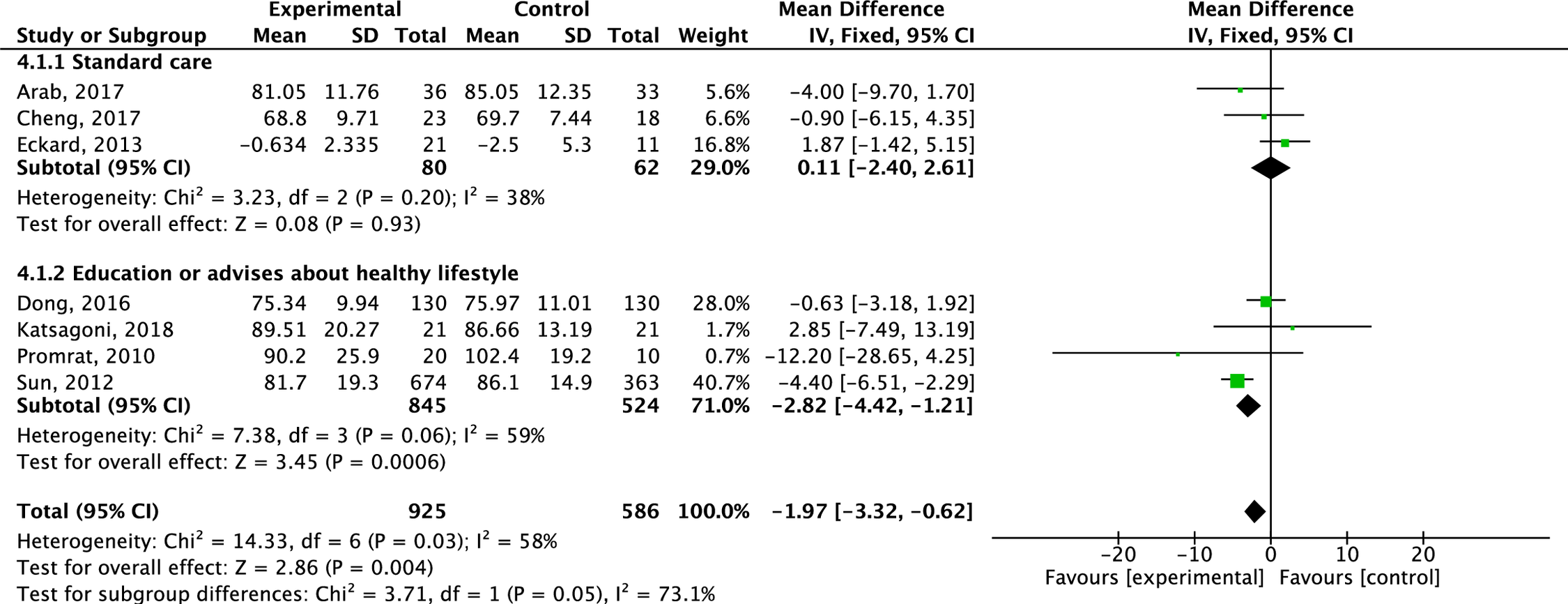

Supplement: S2 Fig — (TIF) [file pone.0263931.s005.tif]

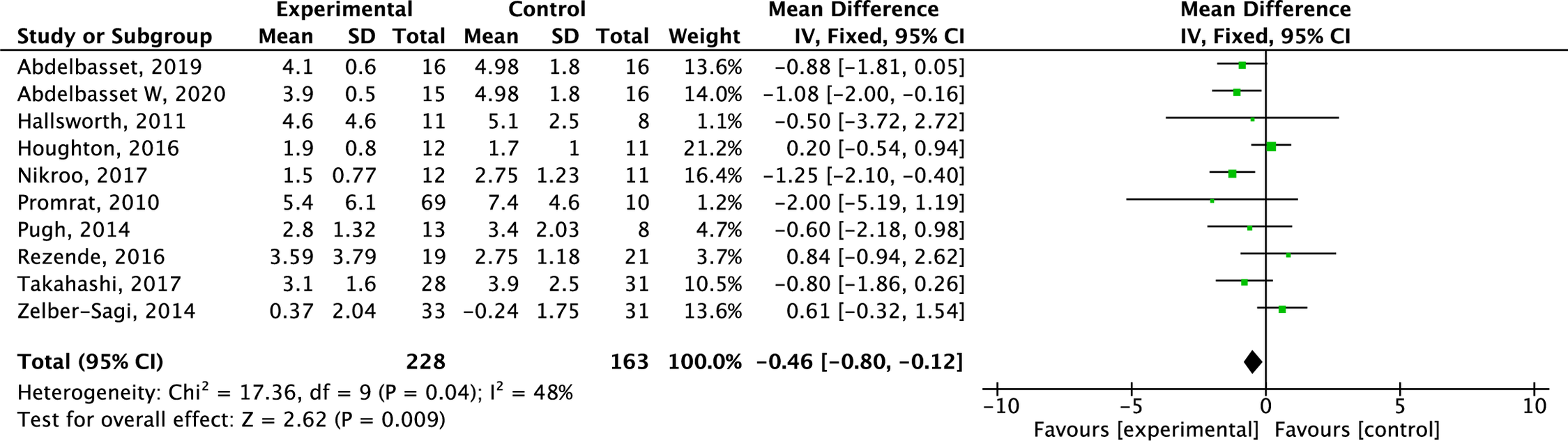

Supplement: S3 Fig — (TIF) [file pone.0263931.s006.tif]

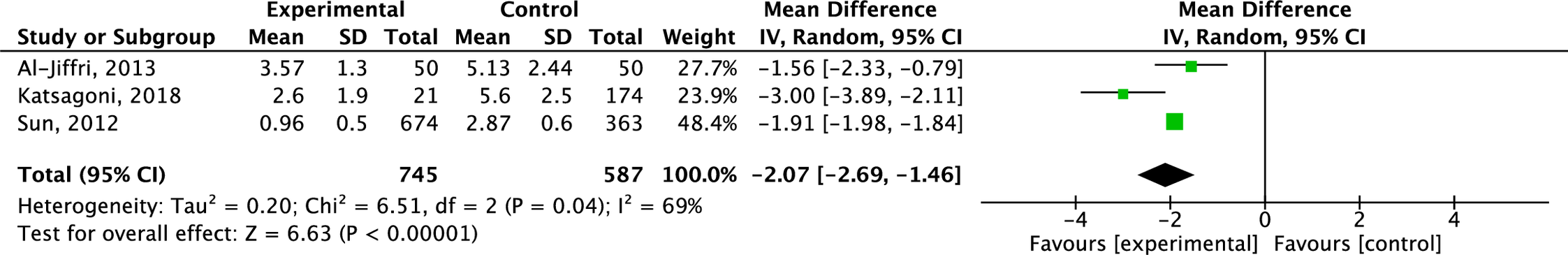

Supplement: S4 Fig — (TIF) [file pone.0263931.s007.tif]

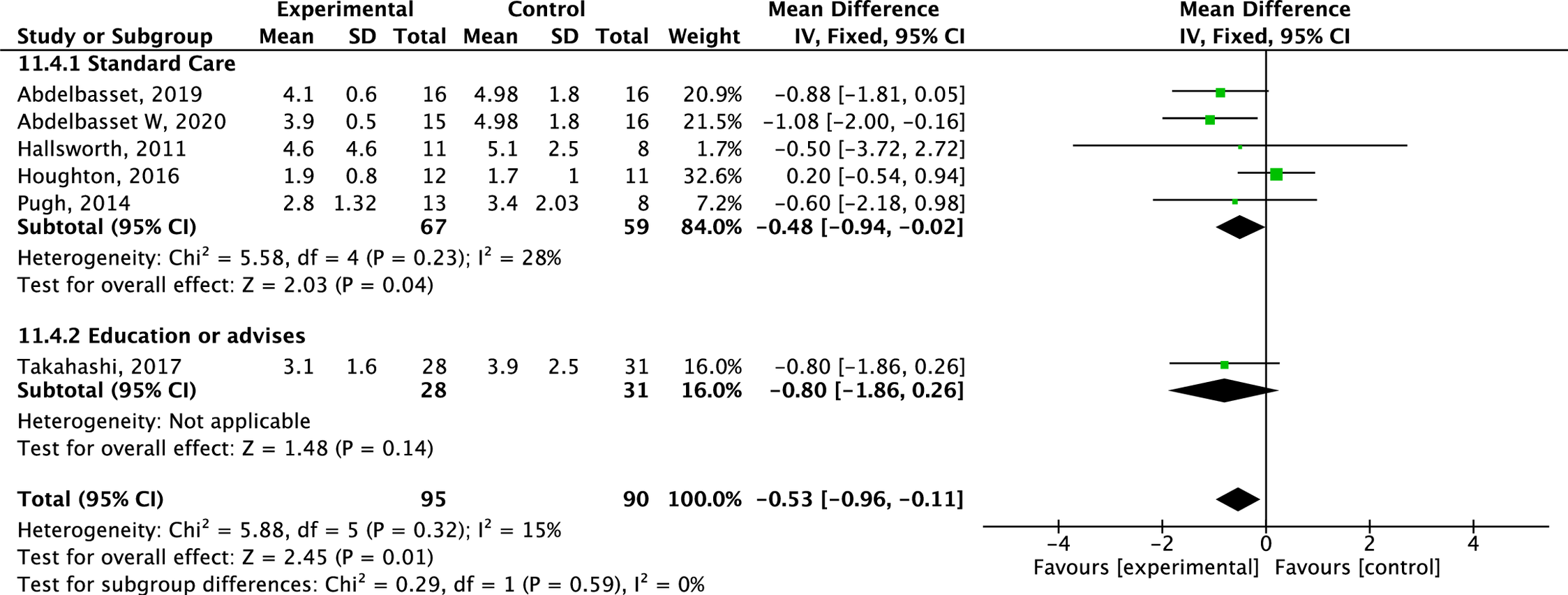

Supplement: S5 Fig — (TIF) [file pone.0263931.s008.tif]

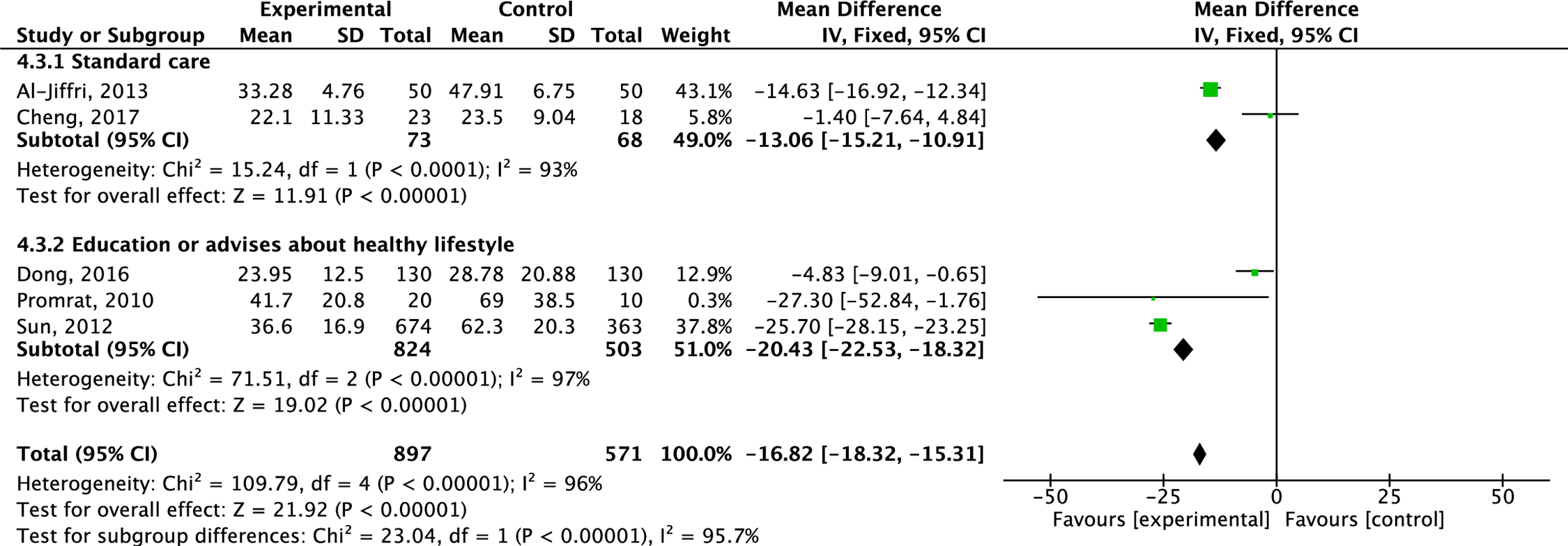

Supplement: S6 Fig — (TIF) [file pone.0263931.s009.tif]

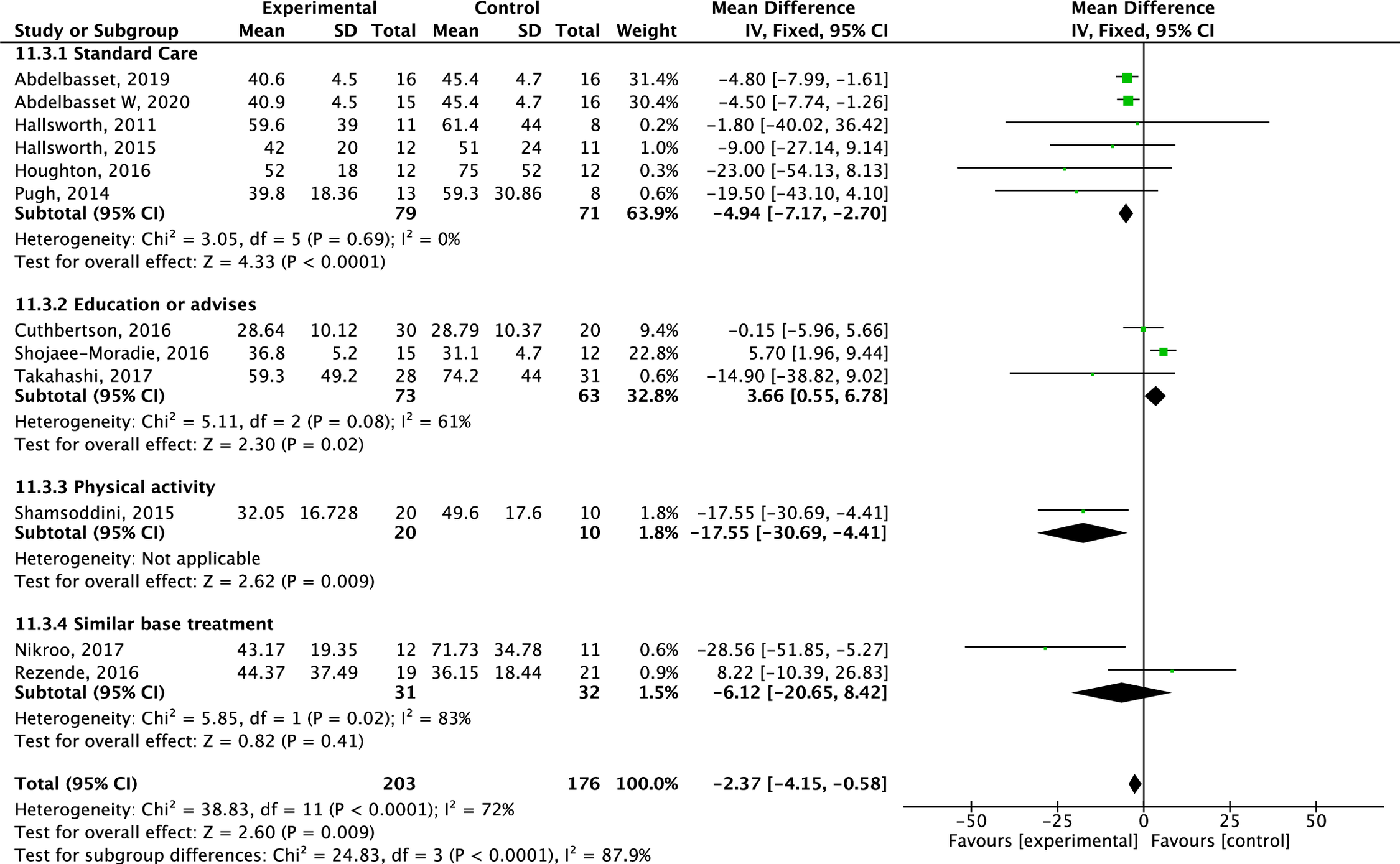

Supplement: S7 Fig — (TIF) [file pone.0263931.s010.tif]

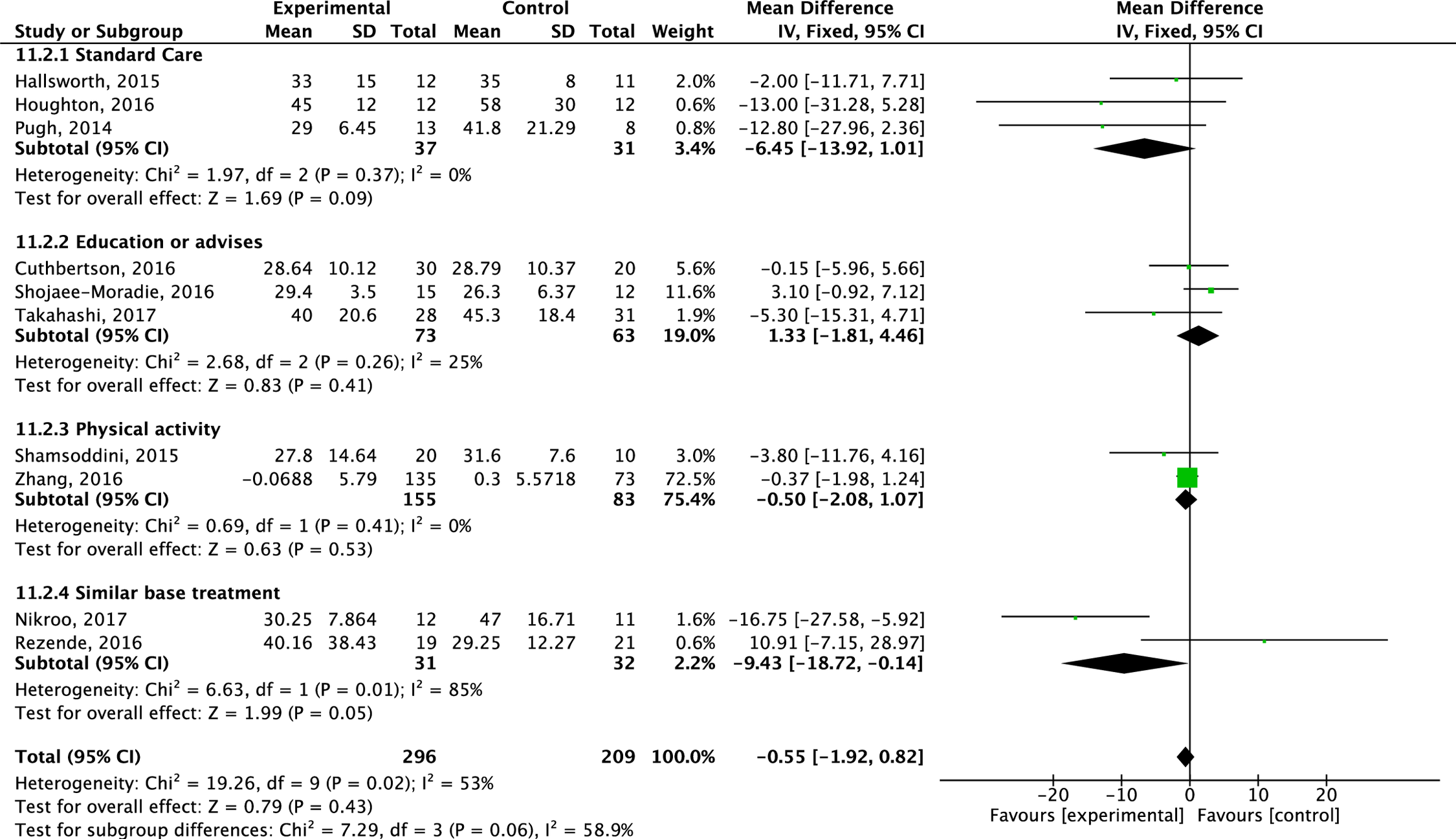

Supplement: S8 Fig — (TIF) [file pone.0263931.s011.tif]

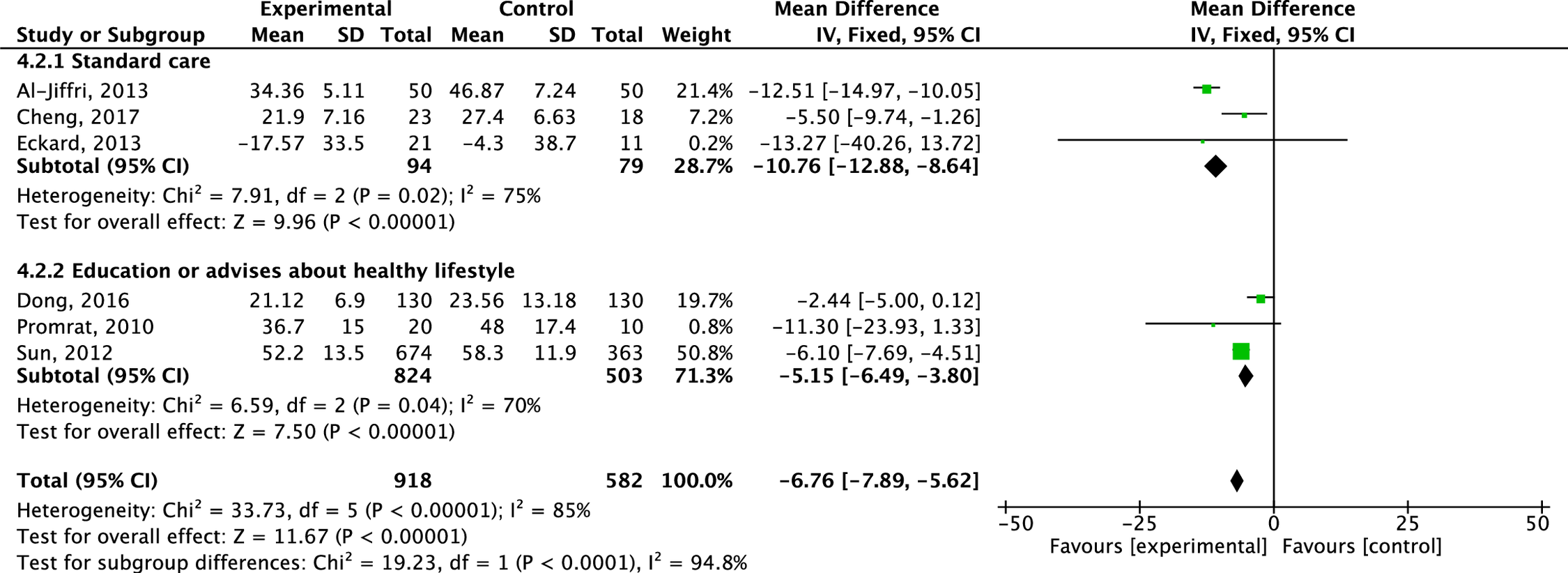

Supplement: S9 Fig — (TIF) [file pone.0263931.s012.tif]

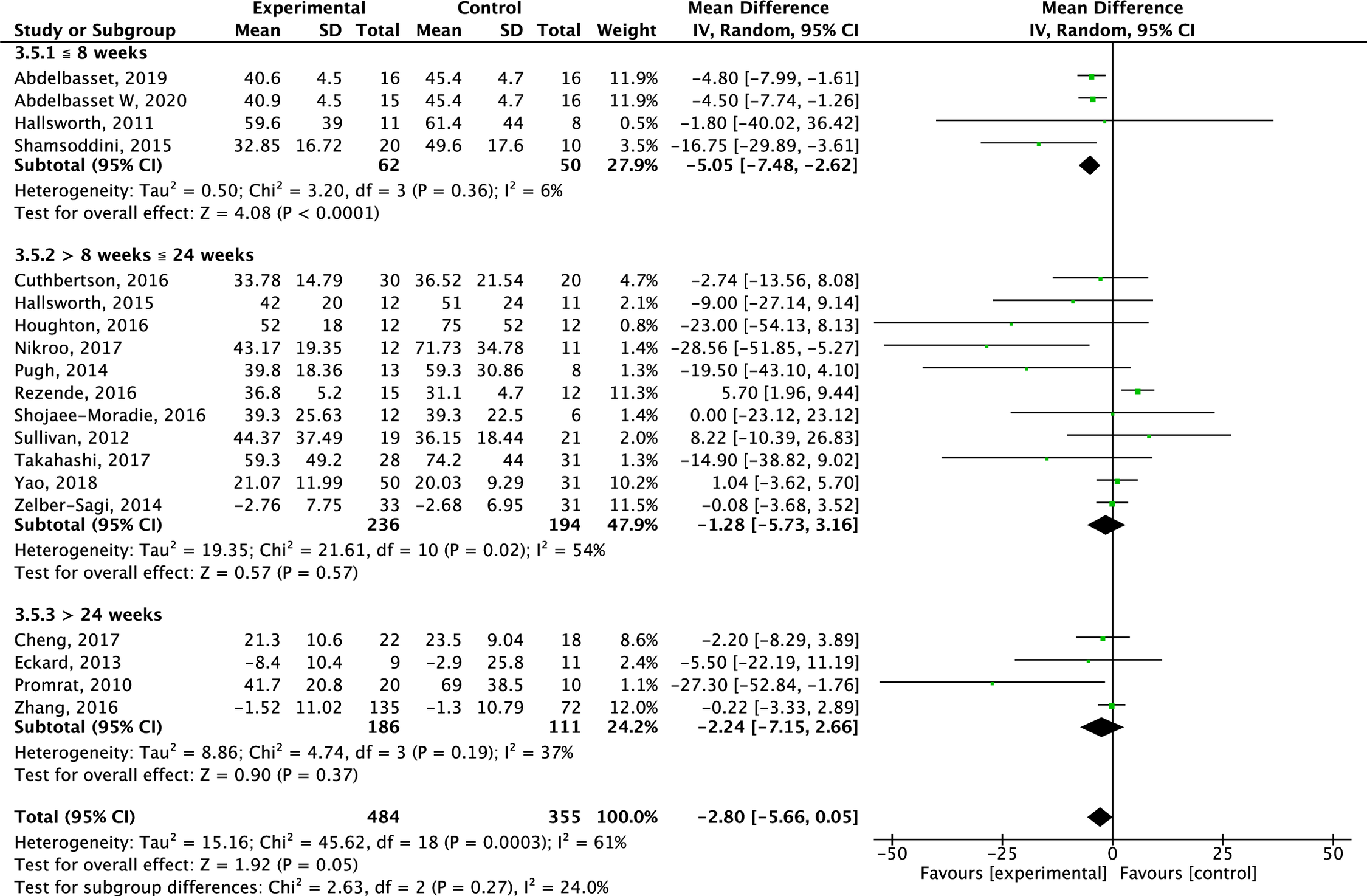

Supplement: S10 Fig — (TIF) [file pone.0263931.s013.tif]

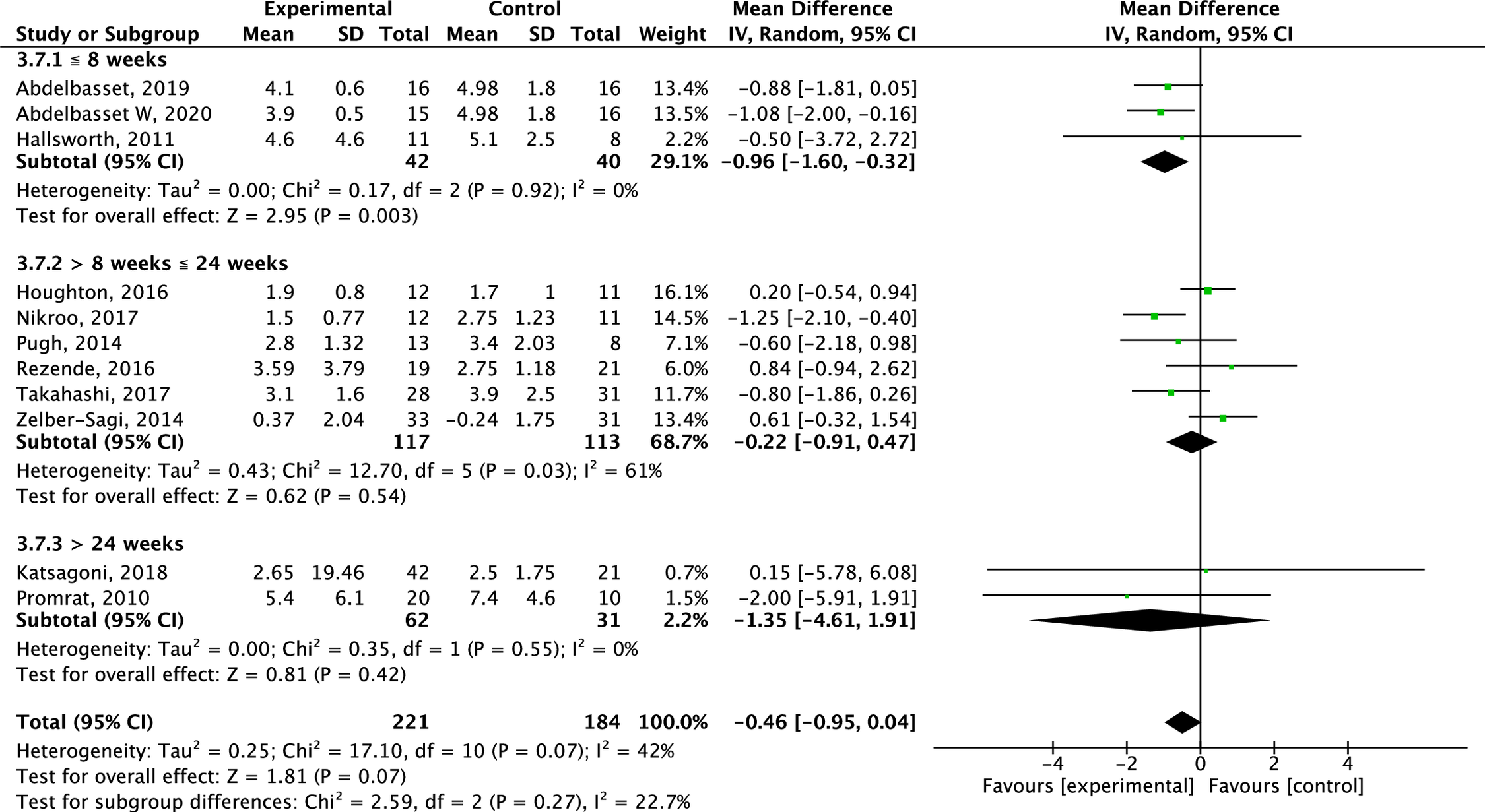

Supplement: S11 Fig — (TIF) [file pone.0263931.s014.tif]

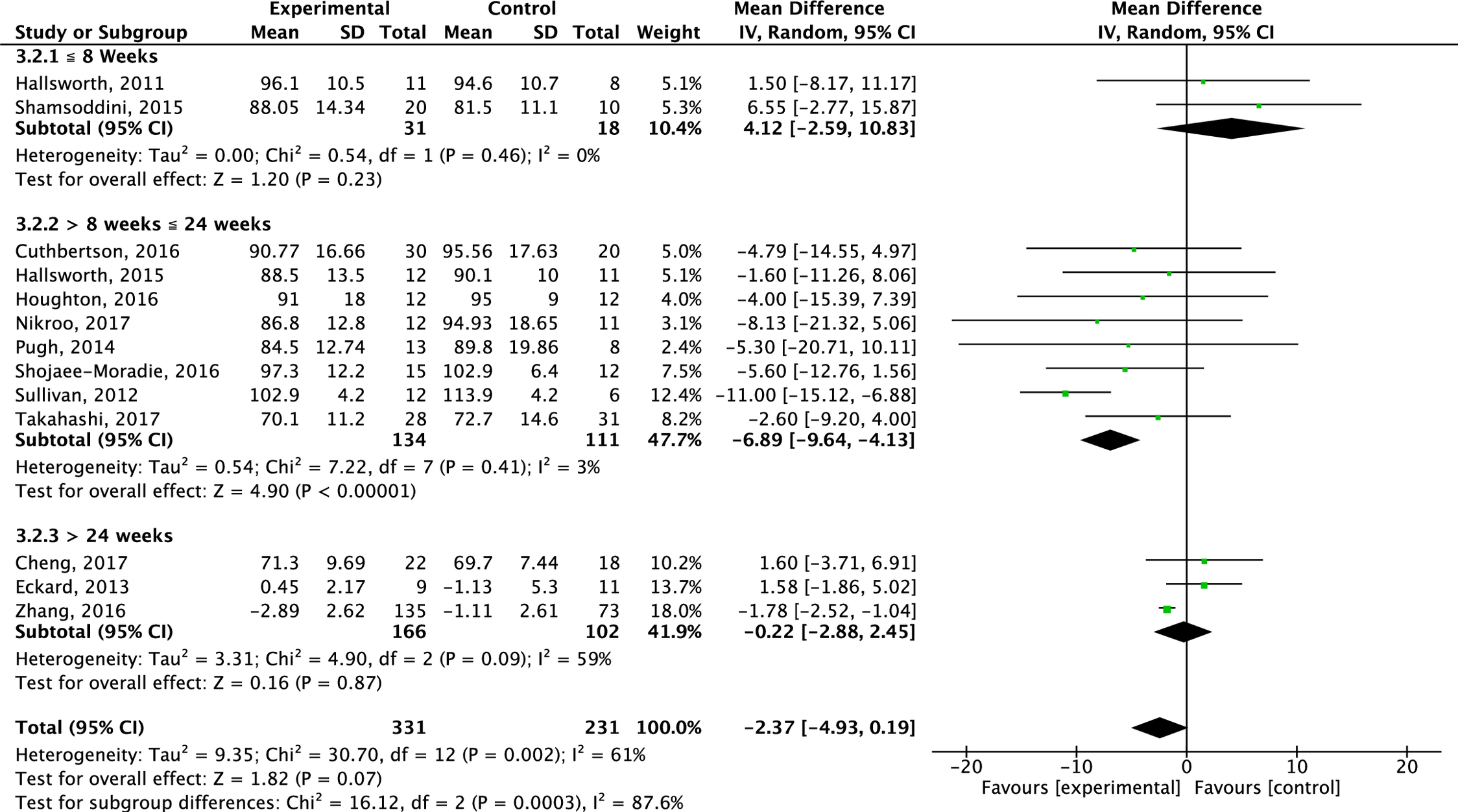

Supplement: S12 Fig — (TIF) [file pone.0263931.s015.tif]

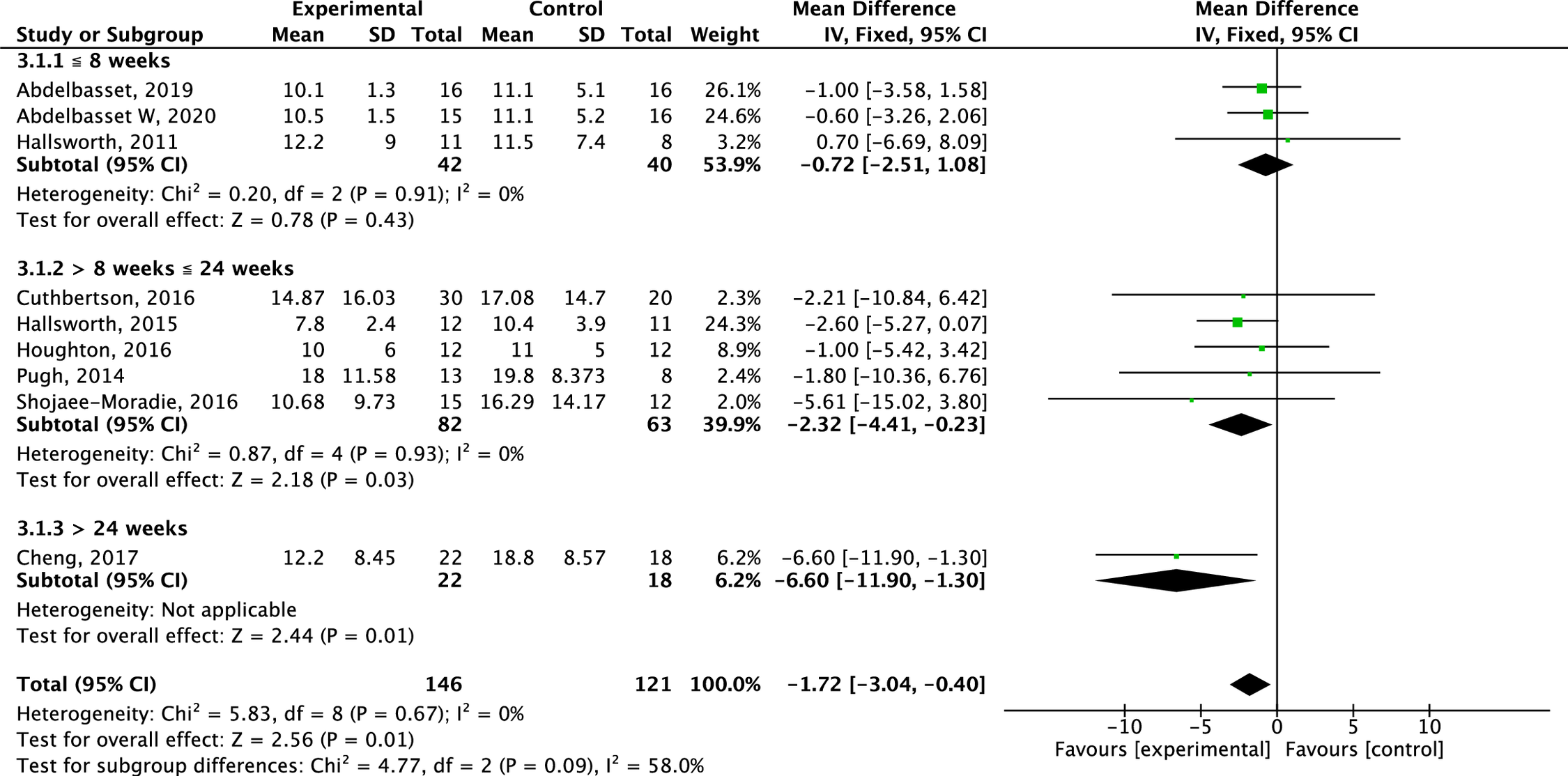

Supplement: S13 Fig — (TIF) [file pone.0263931.s016.tif]

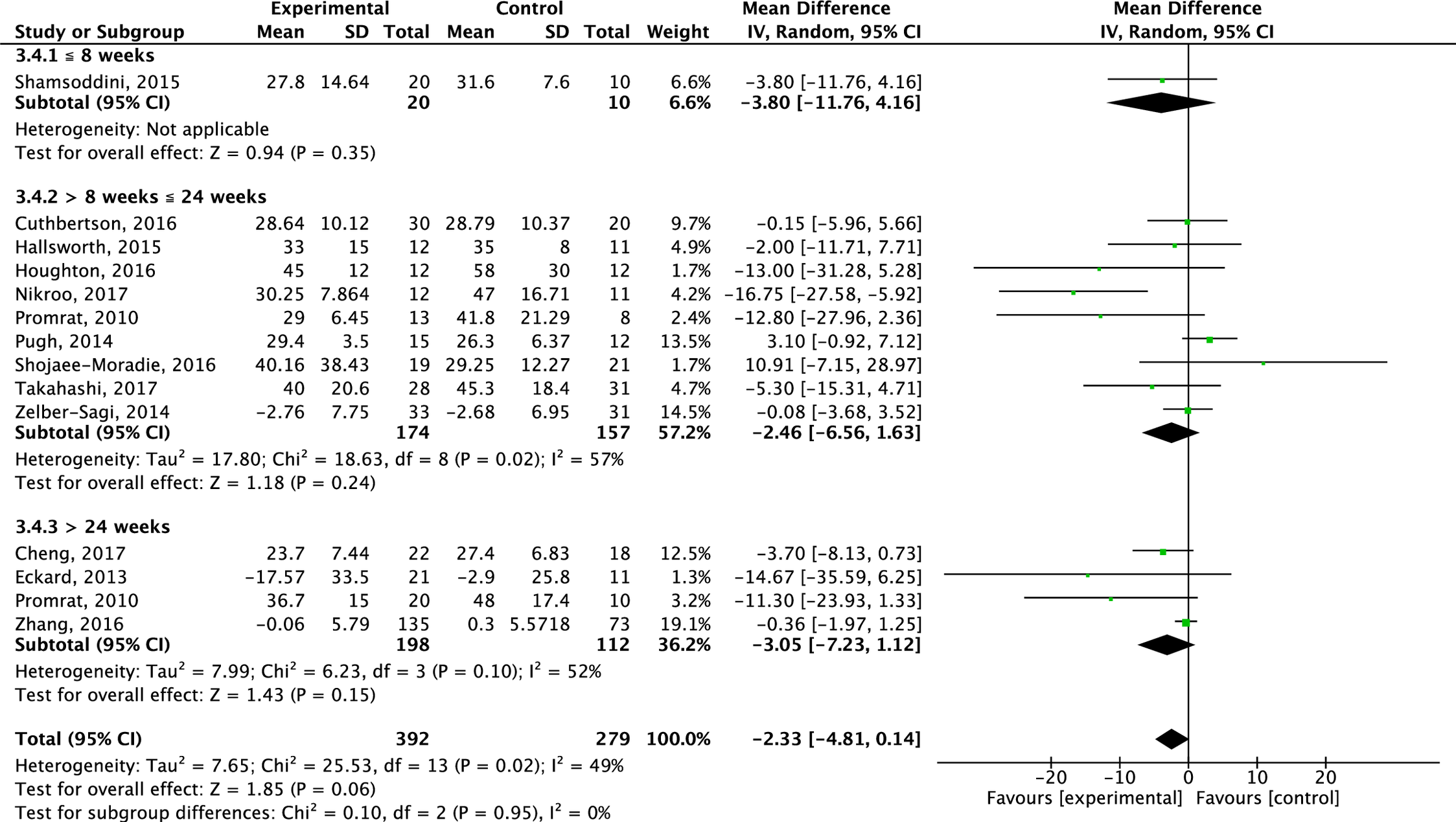

Supplement: S14 Fig — (TIF) [file pone.0263931.s017.tif]

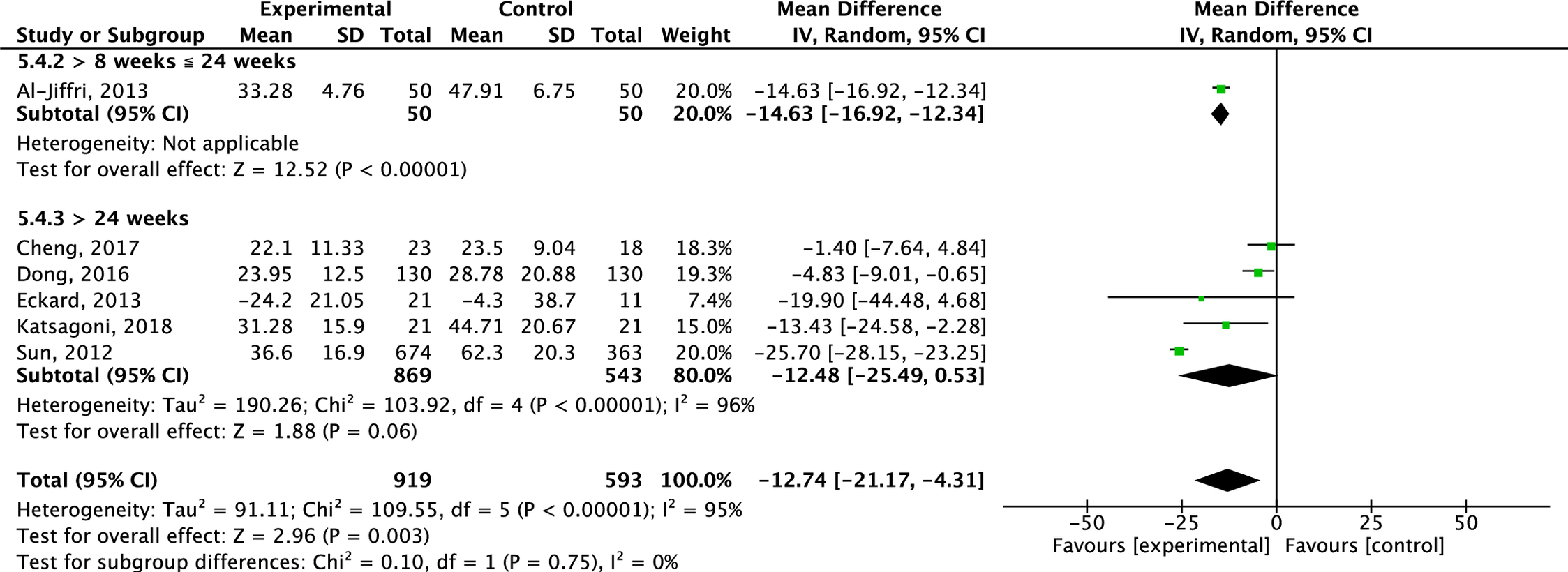

Supplement: S15 Fig — (TIF) [file pone.0263931.s018.tif]

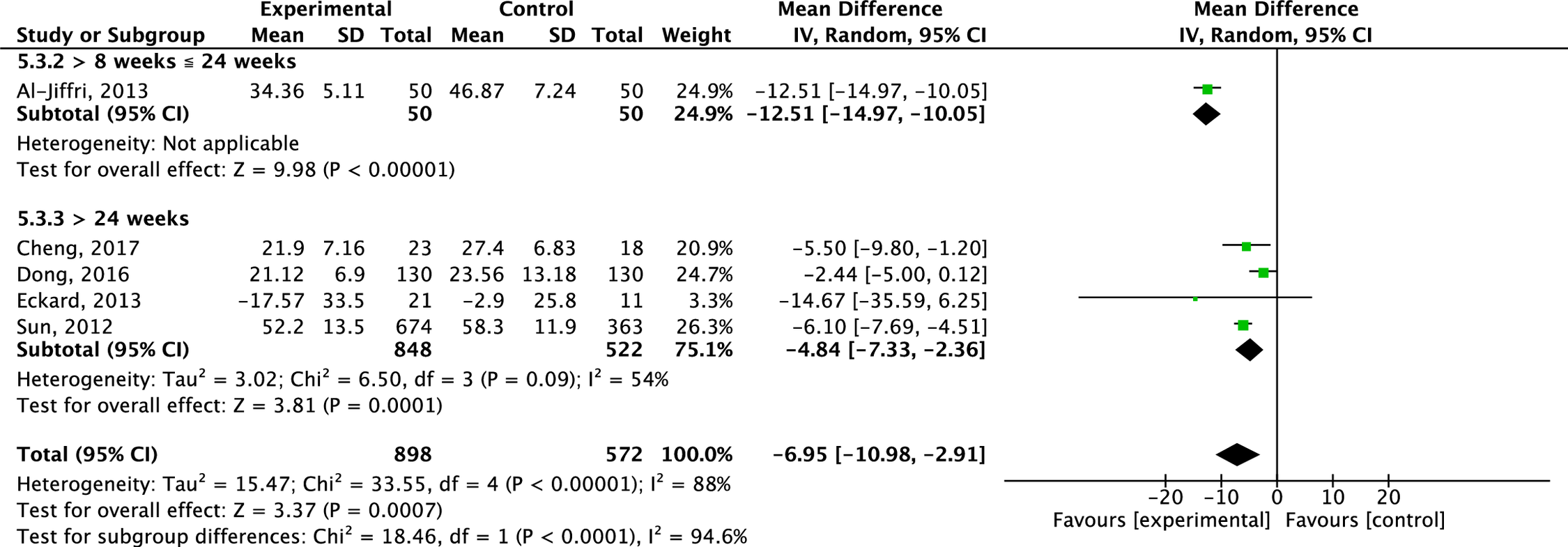

Supplement: S16 Fig — (TIF) [file pone.0263931.s019.tif]

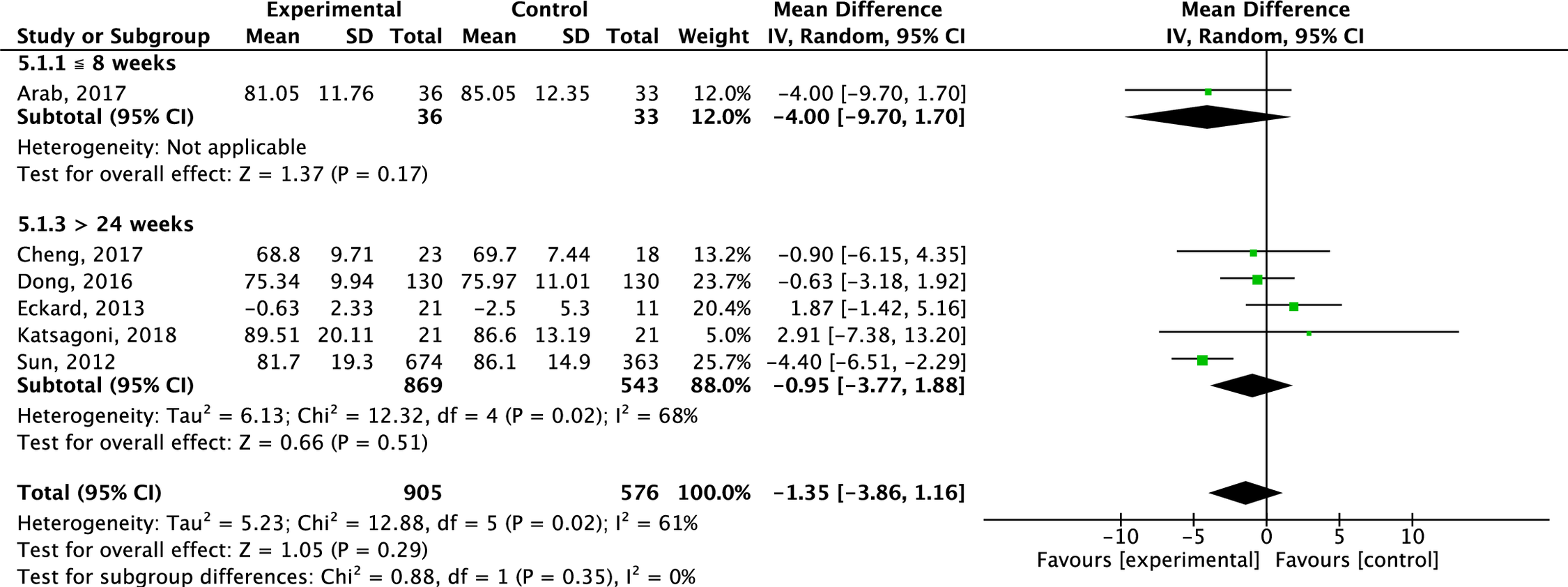

Supplement: S17 Fig — (TIF) [file pone.0263931.s020.tif]

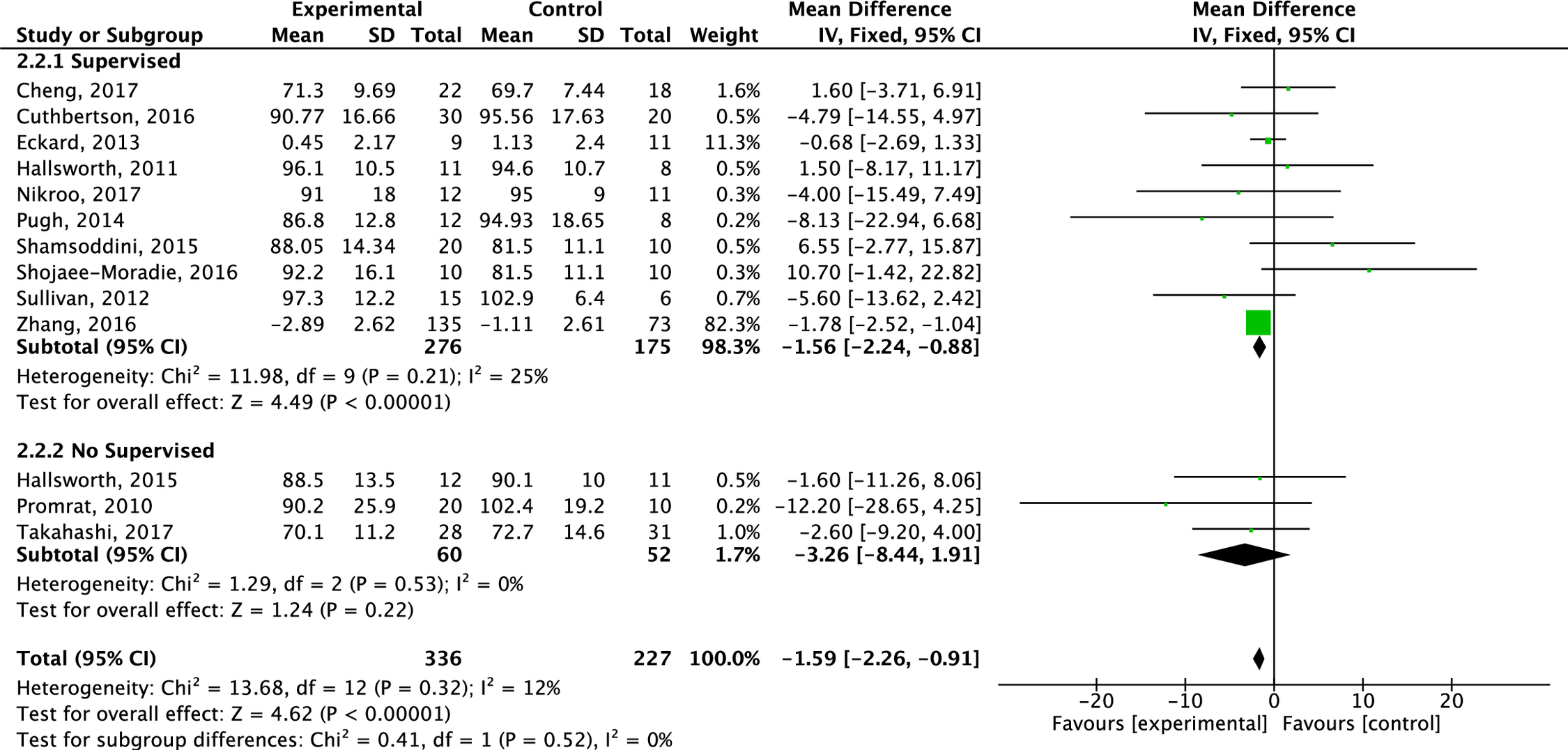

Supplement: S18 Fig — (TIF) [file pone.0263931.s021.tif]

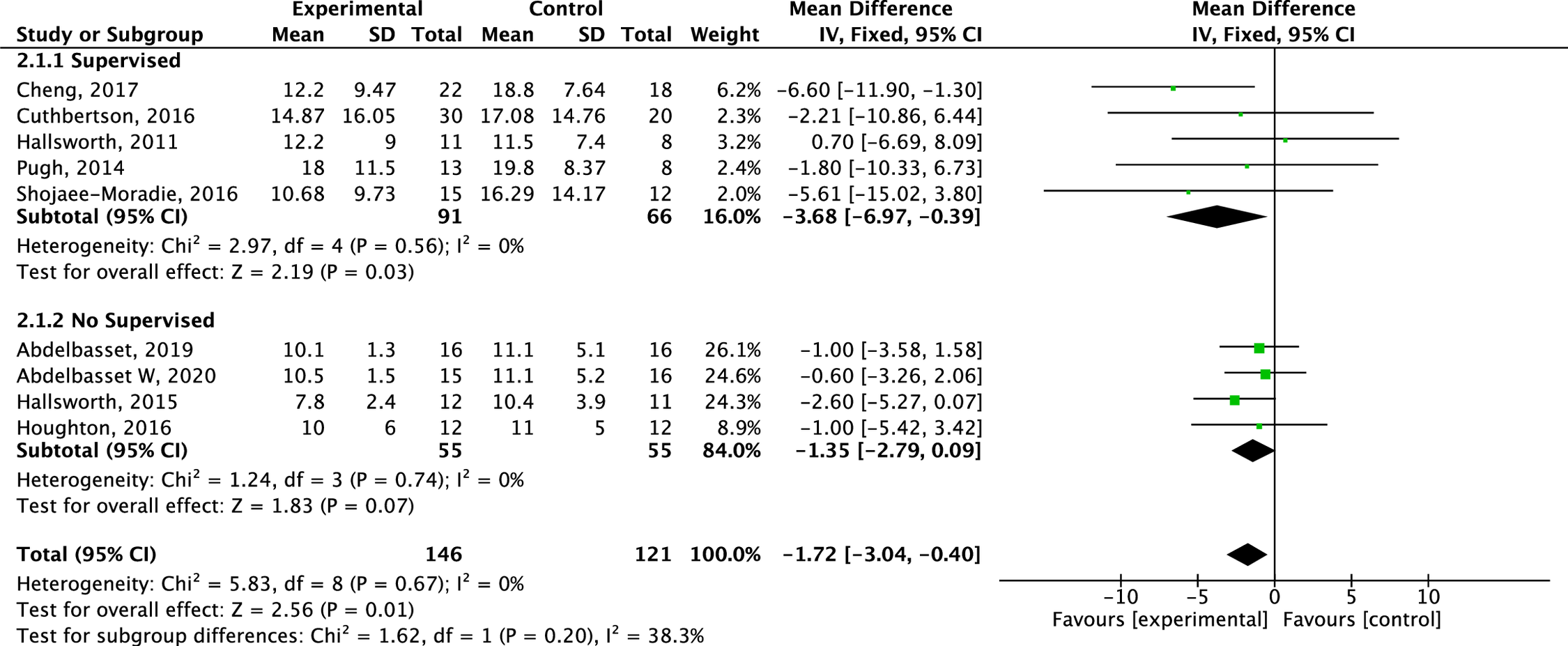

Supplement: S19 Fig — (TIF) [file pone.0263931.s022.tif]

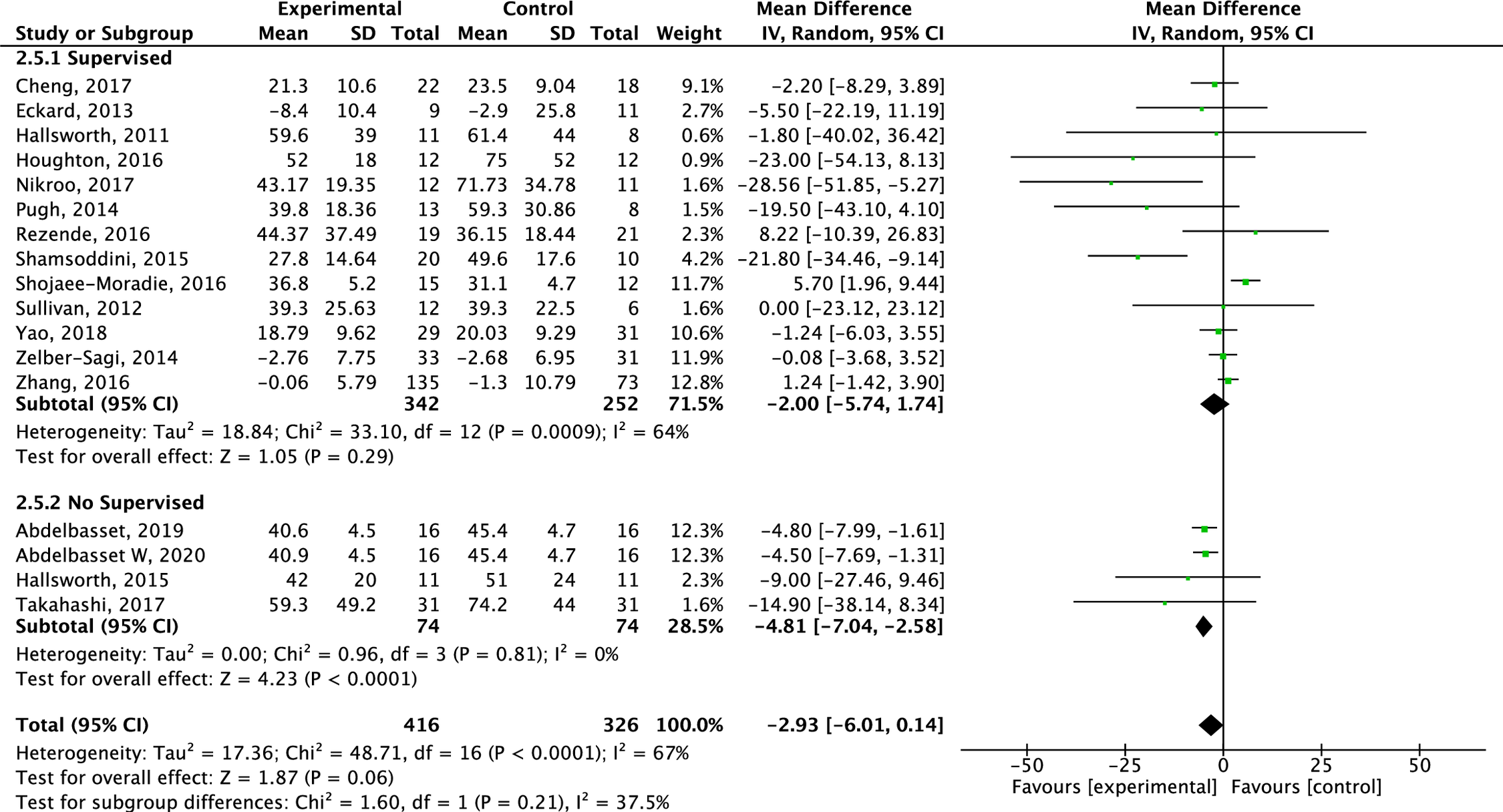

Supplement: S20 Fig — (TIF) [file pone.0263931.s023.tif]

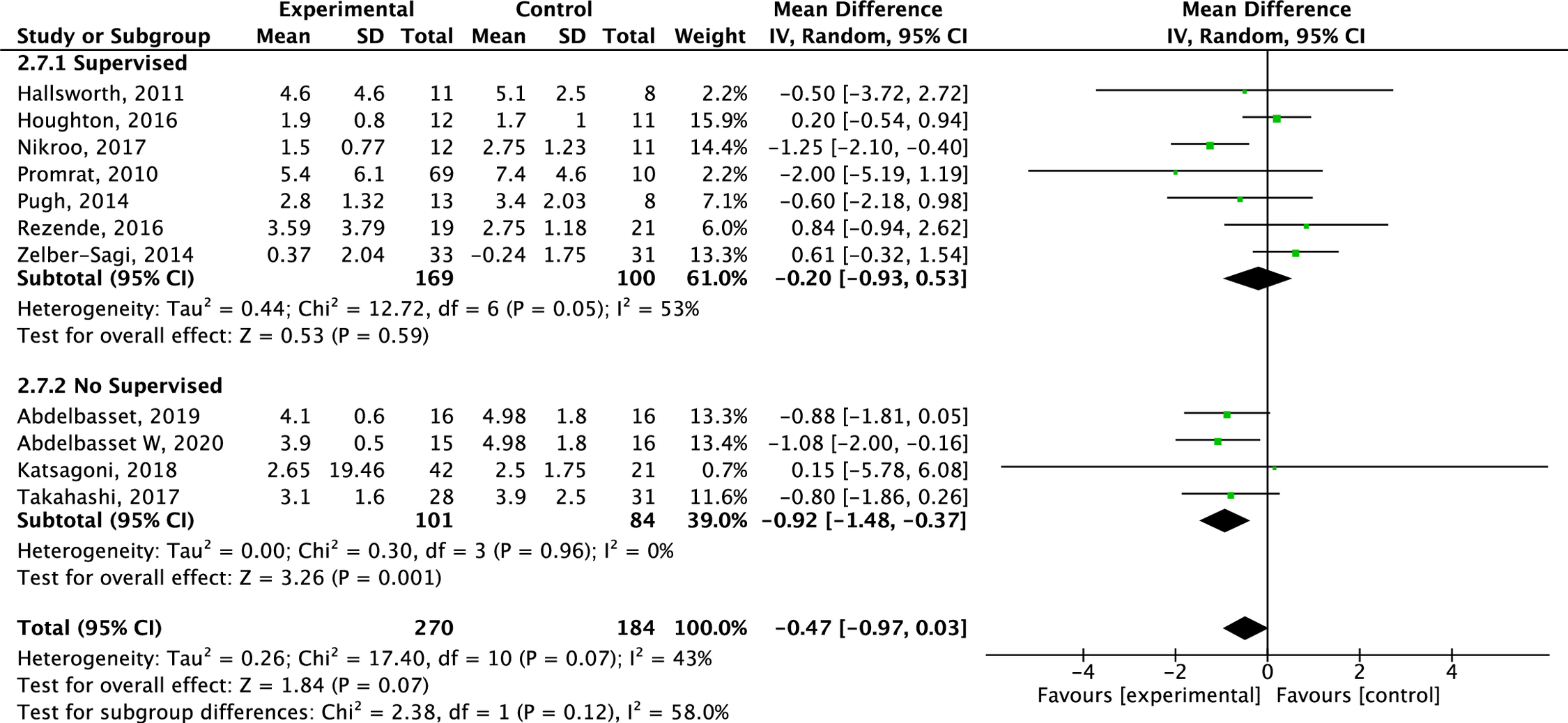

Supplement: S21 Fig — (TIF) [file pone.0263931.s024.tif]

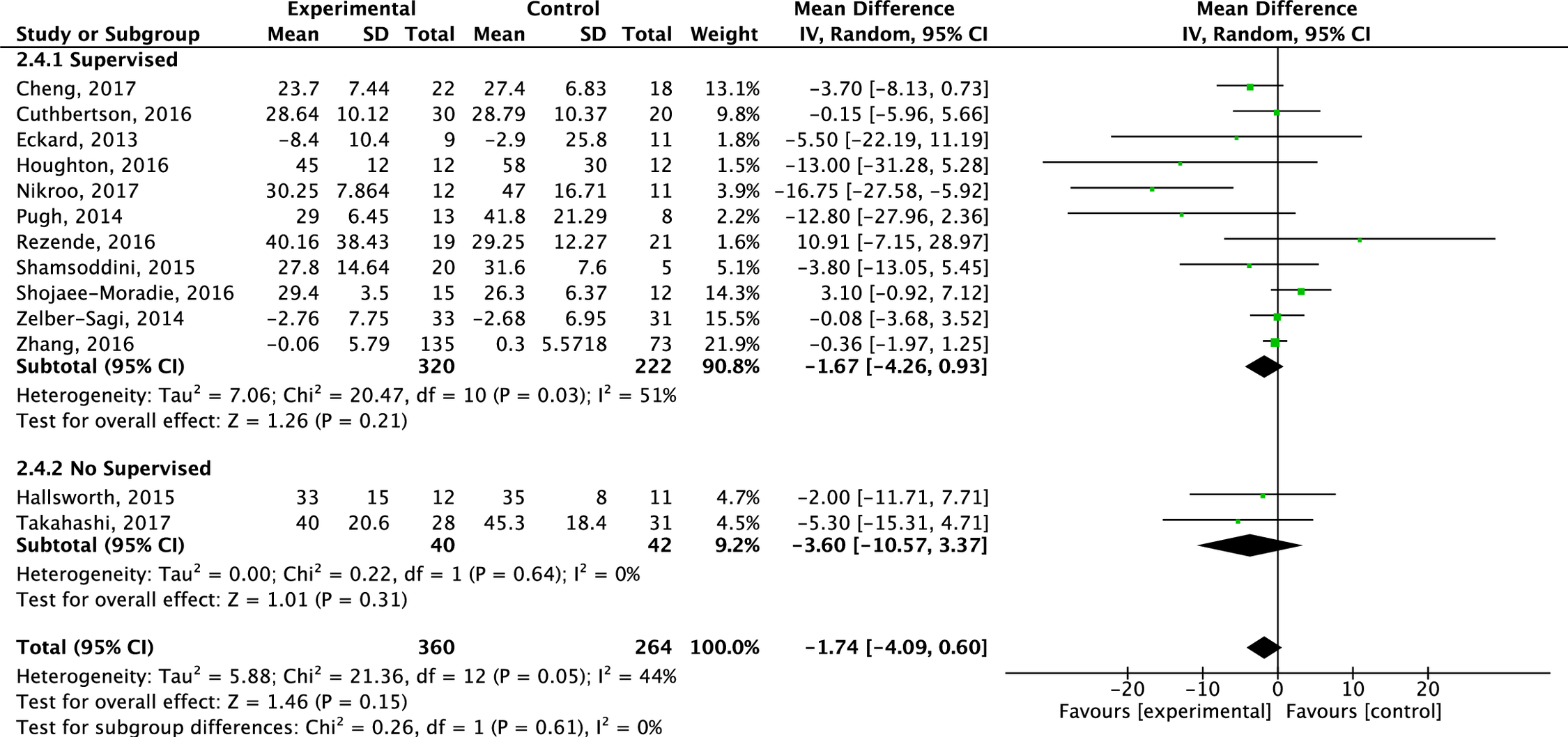

Supplement: S22 Fig — (TIF) [file pone.0263931.s025.tif]
